# Search for supersymmetry in events with a photon, jets, $\mathrm {b}$-jets, and missing transverse momentum in proton–proton collisions at 13$\,\text {Te}\text {V}$

**DOI:** 10.1140/epjc/s10052-019-6926-x

**Published:** 2019-05-25

**Authors:** A. M. Sirunyan, A. Tumasyan, W. Adam, F. Ambrogi, E. Asilar, T. Bergauer, J. Brandstetter, M. Dragicevic, J. Erö, A. Escalante Del Valle, M. Flechl, R. Frühwirth, V. M. Ghete, J. Hrubec, M. Jeitler, N. Krammer, I. Krätschmer, D. Liko, T. Madlener, I. Mikulec, N. Rad, H. Rohringer, J. Schieck, R. Schöfbeck, M. Spanring, D. Spitzbart, W. Waltenberger, J. Wittmann, C.-E. Wulz, M. Zarucki, V. Chekhovsky, V. Mossolov, J. Suarez Gonzalez, E. A. De Wolf, D. Di Croce, X. Janssen, J. Lauwers, M. Pieters, H. Van Haevermaet, P. Van Mechelen, N. Van Remortel, S. Abu Zeid, F. Blekman, J. D’Hondt, J. De Clercq, K. Deroover, G. Flouris, D. Lontkovskyi, S. Lowette, I. Marchesini, S. Moortgat, L. Moreels, Q. Python, K. Skovpen, S. Tavernier, W. Van Doninck, P. Van Mulders, I. Van Parijs, D. Beghin, B. Bilin, H. Brun, B. Clerbaux, G. De Lentdecker, H. Delannoy, B. Dorney, G. Fasanella, L. Favart, R. Goldouzian, A. Grebenyuk, A. K. Kalsi, T. Lenzi, J. Luetic, N. Postiau, E. Starling, L. Thomas, C. Vander Velde, P. Vanlaer, D. Vannerom, Q. Wang, T. Cornelis, D. Dobur, A. Fagot, M. Gul, I. Khvastunov, D. Poyraz, C. Roskas, D. Trocino, M. Tytgat, W. Verbeke, B. Vermassen, M. Vit, N. Zaganidis, H. Bakhshiansohi, O. Bondu, S. Brochet, G. Bruno, C. Caputo, P. David, C. Delaere, M. Delcourt, A. Giammanco, G. Krintiras, V. Lemaitre, A. Magitteri, K. Piotrzkowski, A. Saggio, M. Vidal Marono, P. Vischia, S. Wertz, J. Zobec, F. L. Alves, G. A. Alves, G. Correia Silva, C. Hensel, A. Moraes, M. E. Pol, P. Rebello Teles, E. Belchior Batista Das Chagas, W. Carvalho, J. Chinellato, E. Coelho, E. M. Da Costa, G. G. Da Silveira, D. De Jesus Damiao, C. De Oliveira Martins, S. Fonseca De Souza, H. Malbouisson, D. Matos Figueiredo, M. Melo De Almeida, C. Mora Herrera, L. Mundim, H. Nogima, W. L. Prado Da Silva, L. J. Sanchez Rosas, A. Santoro, A. Sznajder, M. Thiel, E. J. Tonelli Manganote, F. Torres Da Silva De Araujo, A. Vilela Pereira, S. Ahuja, C. A. Bernardes, L. Calligaris, T. R. Fernandez Perez Tomei, E. M. Gregores, P. G. Mercadante, S. F. Novaes, SandraS. Padula, A. Aleksandrov, R. Hadjiiska, P. Iaydjiev, A. Marinov, M. Misheva, M. Rodozov, M. Shopova, G. Sultanov, A. Dimitrov, L. Litov, B. Pavlov, P. Petkov, W. Fang, X. Gao, L. Yuan, M. Ahmad, J. G. Bian, G. M. Chen, H. S. Chen, M. Chen, Y. Chen, C. H. Jiang, D. Leggat, H. Liao, Z. Liu, S. M. Shaheen, A. Spiezia, J. Tao, E. Yazgan, H. Zhang, S. Zhang, J. Zhao, Y. Ban, G. Chen, A. Levin, J. Li, L. Li, Q. Li, Y. Mao, S. J. Qian, D. Wang, Y. Wang, C. Avila, A. Cabrera, C. A. Carrillo Montoya, L. F. Chaparro Sierra, C. Florez, C. F. González Hernández, M. A. Segura Delgado, B. Courbon, N. Godinovic, D. Lelas, I. Puljak, T. Sculac, Z. Antunovic, M. Kovac, V. Brigljevic, D. Ferencek, K. Kadija, B. Mesic, M. Roguljic, A. Starodumov, T. Susa, M. W. Ather, A. Attikis, M. Kolosova, G. Mavromanolakis, J. Mousa, C. Nicolaou, F. Ptochos, P. A. Razis, H. Rykaczewski, M. Finger, M. Finger, E. Ayala, E. Carrera Jarrin, A. Ellithi Kamel, S. Khalil, E. Salama, S. Bhowmik, A. Carvalho Antunes De Oliveira, R. K. Dewanjee, K. Ehataht, M. Kadastik, M. Raidal, C. Veelken, P. Eerola, H. Kirschenmann, J. Pekkanen, M. Voutilainen, J. Havukainen, J. K. Heikkilä, T. Järvinen, V. Karimäki, R. Kinnunen, T. Lampén, K. Lassila-Perini, S. Laurila, S. Lehti, T. Lindén, P. Luukka, T. Mäenpää, H. Siikonen, E. Tuominen, J. Tuominiemi, T. Tuuva, M. Besancon, F. Couderc, M. Dejardin, D. Denegri, J. L. Faure, F. Ferri, S. Ganjour, A. Givernaud, P. Gras, G. Hamel de Monchenault, P. Jarry, C. Leloup, E. Locci, J. Malcles, G. Negro, J. Rander, A. Rosowsky, M. Ö. Sahin, M. Titov, A. Abdulsalam, C. Amendola, I. Antropov, F. Beaudette, P. Busson, C. Charlot, R. Granier de Cassagnac, I. Kucher, A. Lobanov, J. Martin Blanco, C. Martin Perez, M. Nguyen, C. Ochando, G. Ortona, P. Paganini, J. Rembser, R. Salerno, J. B. Sauvan, Y. Sirois, A. G. Stahl Leiton, A. Zabi, A. Zghiche, J.-L. Agram, J. Andrea, D. Bloch, J.-M. Brom, E. C. Chabert, V. Cherepanov, C. Collard, E. Conte, J.-C. Fontaine, D. Gelé, U. Goerlach, M. Jansová, A.-C. Le Bihan, N. Tonon, P. Van Hove, S. Gadrat, S. Beauceron, C. Bernet, G. Boudoul, N. Chanon, R. Chierici, D. Contardo, P. Depasse, H. El Mamouni, J. Fay, L. Finco, S. Gascon, M. Gouzevitch, G. Grenier, B. Ille, F. Lagarde, I. B. Laktineh, H. Lattaud, M. Lethuillier, L. Mirabito, S. Perries, A. Popov, V. Sordini, G. Touquet, M. Vander Donckt, S. Viret, T. Toriashvili, Z. Tsamalaidze, C. Autermann, L. Feld, M. K. Kiesel, K. Klein, M. Lipinski, M. Preuten, M. P. Rauch, C. Schomakers, J. Schulz, M. Teroerde, B. Wittmer, A. Albert, D. Duchardt, M. Erdmann, S. Erdweg, T. Esch, R. Fischer, S. Ghosh, A. Güth, T. Hebbeker, C. Heidemann, K. Hoepfner, H. Keller, L. Mastrolorenzo, M. Merschmeyer, A. Meyer, P. Millet, S. Mukherjee, T. Pook, M. Radziej, H. Reithler, M. Rieger, A. Schmidt, D. Teyssier, S. Thüer, G. Flügge, O. Hlushchenko, T. Kress, T. Müller, A. Nehrkorn, A. Nowack, C. Pistone, O. Pooth, D. Roy, H. Sert, A. Stahl, M. Aldaya Martin, T. Arndt, C. Asawatangtrakuldee, I. Babounikau, K. Beernaert, O. Behnke, U. Behrens, A. Bermúdez Martínez, D. Bertsche, A. A. Bin Anuar, K. Borras, V. Botta, A. Campbell, P. Connor, C. Contreras-Campana, V. Danilov, A. De Wit, M. M. Defranchis, C. Diez Pardos, D. Domínguez Damiani, G. Eckerlin, T. Eichhorn, A. Elwood, E. Eren, E. Gallo, A. Geiser, J. M. Grados Luyando, A. Grohsjean, M. Guthoff, M. Haranko, A. Harb, H. Jung, M. Kasemann, J. Keaveney, C. Kleinwort, J. Knolle, D. Krücker, W. Lange, A. Lelek, T. Lenz, J. Leonard, K. Lipka, W. Lohmann, R. Mankel, I.-A. Melzer-Pellmann, A. B. Meyer, M. Meyer, M. Missiroli, G. Mittag, J. Mnich, V. Myronenko, S. K. Pflitsch, D. Pitzl, A. Raspereza, M. Savitskyi, P. Saxena, P. Schütze, C. Schwanenberger, R. Shevchenko, A. Singh, H. Tholen, O. Turkot, A. Vagnerini, M. Van De Klundert, G. P. Van Onsem, R. Walsh, Y. Wen, K. Wichmann, C. Wissing, O. Zenaiev, R. Aggleton, S. Bein, L. Benato, A. Benecke, T. Dreyer, A. Ebrahimi, E. Garutti, D. Gonzalez, P. Gunnellini, J. Haller, A. Hinzmann, A. Karavdina, G. Kasieczka, R. Klanner, R. Kogler, N. Kovalchuk, S. Kurz, V. Kutzner, J. Lange, D. Marconi, J. Multhaup, M. Niedziela, C. E. N. Niemeyer, D. Nowatschin, A. Perieanu, A. Reimers, O. Rieger, C. Scharf, P. Schleper, S. Schumann, J. Schwandt, J. Sonneveld, H. Stadie, G. Steinbrück, F. M. Stober, M. Stöver, B. Vormwald, I. Zoi, M. Akbiyik, C. Barth, M. Baselga, S. Baur, E. Butz, R. Caspart, T. Chwalek, F. Colombo, W. De Boer, A. Dierlamm, K. El Morabit, N. Faltermann, B. Freund, M. Giffels, M. A. Harrendorf, F. Hartmann, S. M. Heindl, U. Husemann, I. Katkov, S. Kudella, S. Mitra, M. U. Mozer, Th. Müller, M. Musich, M. Plagge, G. Quast, K. Rabbertz, M. Schröder, I. Shvetsov, H. J. Simonis, R. Ulrich, S. Wayand, M. Weber, T. Weiler, C. Wöhrmann, R. Wolf, G. Anagnostou, G. Daskalakis, T. Geralis, A. Kyriakis, D. Loukas, G. Paspalaki, A. Agapitos, G. Karathanasis, P. Kontaxakis, A. Panagiotou, I. Papavergou, N. Saoulidou, E. Tziaferi, K. Vellidis, K. Kousouris, I. Papakrivopoulos, G. Tsipolitis, I. Evangelou, C. Foudas, P. Gianneios, P. Katsoulis, P. Kokkas, S. Mallios, N. Manthos, I. Papadopoulos, E. Paradas, J. Strologas, F. A. Triantis, D. Tsitsonis, M. Bartók, M. Csanad, N. Filipovic, P. Major, M. I. Nagy, G. Pasztor, O. Surányi, G. I. Veres, G. Bencze, C. Hajdu, D. Horvath, Á. Hunyadi, F. Sikler, T. Á. Vámi, V. Veszpremi, G. Vesztergombi, N. Beni, S. Czellar, J. Karancsi, A. Makovec, J. Molnar, Z. Szillasi, P. Raics, Z. L. Trocsanyi, B. Ujvari, S. Choudhury, J. R. Komaragiri, P. C. Tiwari, S. Bahinipati, C. Kar, P. Mal, K. Mandal, A. Nayak, S. Roy Chowdhury, D. K. Sahoo, S. K. Swain, S. Bansal, S. B. Beri, V. Bhatnagar, S. Chauhan, R. Chawla, N. Dhingra, R. Gupta, A. Kaur, M. Kaur, S. Kaur, P. Kumari, M. Lohan, M. Meena, A. Mehta, K. Sandeep, S. Sharma, J. B. Singh, A. K. Virdi, G. Walia, A. Bhardwaj, B. C. Choudhary, R. B. Garg, M. Gola, S. Keshri, Ashok Kumar, S. Malhotra, M. Naimuddin, P. Priyanka, K. Ranjan, Aashaq Shah, R. Sharma, R. Bhardwaj, M. Bharti, R. Bhattacharya, S. Bhattacharya, U. Bhawandeep, D. Bhowmik, S. Dey, S. Dutt, S. Dutta, S. Ghosh, M. Maity, K. Mondal, S. Nandan, A. Purohit, P. K. Rout, A. Roy, G. Saha, S. Sarkar, T. Sarkar, M. Sharan, B. Singh, S. Thakur, P. K. Behera, A. Muhammad, R. Chudasama, D. Dutta, V. Jha, V. Kumar, D. K. Mishra, P. K. Netrakanti, L. M. Pant, P. Shukla, P. Suggisetti, T. Aziz, M. A. Bhat, S. Dugad, G. B. Mohanty, N. Sur, RavindraKumar Verma, S. Banerjee, S. Bhattacharya, S. Chatterjee, P. Das, M. Guchait, Sa. Jain, S. Karmakar, S. Kumar, G. Majumder, K. Mazumdar, N. Sahoo, S. Chauhan, S. Dube, V. Hegde, A. Kapoor, K. Kothekar, S. Pandey, A. Rane, A. Rastogi, S. Sharma, S. Chenarani, E. Eskandari Tadavani, S. M. Etesami, M. Khakzad, M. Mohammadi Najafabadi, M. Naseri, F. Rezaei Hosseinabadi, B. Safarzadeh, M. Zeinali, M. Felcini, M. Grunewald, M. Abbrescia, C. Calabria, A. Colaleo, D. Creanza, L. Cristella, N. De Filippis, M. De Palma, A. Di Florio, F. Errico, L. Fiore, A. Gelmi, G. Iaselli, M. Ince, S. Lezki, G. Maggi, M. Maggi, G. Miniello, S. My, S. Nuzzo, A. Pompili, G. Pugliese, R. Radogna, A. Ranieri, G. Selvaggi, A. Sharma, L. Silvestris, R. Venditti, P. Verwilligen, G. Abbiendi, C. Battilana, D. Bonacorsi, L. Borgonovi, S. Braibant-Giacomelli, R. Campanini, P. Capiluppi, A. Castro, F. R. Cavallo, S. S. Chhibra, G. Codispoti, M. Cuffiani, G. M. Dallavalle, F. Fabbri, A. Fanfani, E. Fontanesi, P. Giacomelli, C. Grandi, L. Guiducci, F. Iemmi, S. Lo Meo, S. Marcellini, G. Masetti, A. Montanari, F. L. Navarria, A. Perrotta, F. Primavera, A. M. Rossi, T. Rovelli, G. P. Siroli, N. Tosi, S. Albergo, A. Di Mattia, R. Potenza, A. Tricomi, C. Tuve, G. Barbagli, K. Chatterjee, V. Ciulli, C. Civinini, R. D’Alessandro, E. Focardi, G. Latino, P. Lenzi, M. Meschini, S. Paoletti, L. Russo, G. Sguazzoni, D. Strom, L. Viliani, L. Benussi, S. Bianco, F. Fabbri, D. Piccolo, F. Ferro, R. Mulargia, E. Robutti, S. Tosi, A. Benaglia, A. Beschi, F. Brivio, V. Ciriolo, S. Di Guida, M. E. Dinardo, S. Fiorendi, S. Gennai, A. Ghezzi, P. Govoni, M. Malberti, S. Malvezzi, D. Menasce, F. Monti, L. Moroni, M. Paganoni, D. Pedrini, S. Ragazzi, T. Tabarelli de Fatis, D. Zuolo, S. Buontempo, N. Cavallo, A. De Iorio, A. Di Crescenzo, F. Fabozzi, F. Fienga, G. Galati, A. O. M. Iorio, L. Lista, S. Meola, P. Paolucci, C. Sciacca, E. Voevodina, P. Azzi, N. Bacchetta, D. Bisello, A. Boletti, A. Bragagnolo, R. Carlin, P. Checchia, M. Dall’Osso, P. De Castro Manzano, T. Dorigo, U. Dosselli, F. Gasparini, U. Gasparini, A. Gozzelino, S. Y. Hoh, S. Lacaprara, P. Lujan, M. Margoni, A. T. Meneguzzo, J. Pazzini, M. Presilla, P. Ronchese, R. Rossin, F. Simonetto, A. Tiko, E. Torassa, M. Tosi, M. Zanetti, P. Zotto, G. Zumerle, A. Braghieri, A. Magnani, P. Montagna, S. P. Ratti, V. Re, M. Ressegotti, C. Riccardi, P. Salvini, I. Vai, P. Vitulo, M. Biasini, G. M. Bilei, C. Cecchi, D. Ciangottini, L. Fanò, P. Lariccia, R. Leonardi, E. Manoni, G. Mantovani, V. Mariani, M. Menichelli, A. Rossi, A. Santocchia, D. Spiga, K. Androsov, P. Azzurri, G. Bagliesi, L. Bianchini, T. Boccali, L. Borrello, R. Castaldi, M. A. Ciocci, R. Dell’Orso, G. Fedi, F. Fiori, L. Giannini, A. Giassi, M. T. Grippo, F. Ligabue, E. Manca, G. Mandorli, A. Messineo, F. Palla, A. Rizzi, G. Rolandi, P. Spagnolo, R. Tenchini, G. Tonelli, A. Venturi, P. G. Verdini, L. Barone, F. Cavallari, M. Cipriani, D. Del Re, E. Di Marco, M. Diemoz, S. Gelli, E. Longo, B. Marzocchi, P. Meridiani, G. Organtini, F. Pandolfi, R. Paramatti, F. Preiato, S. Rahatlou, C. Rovelli, F. Santanastasio, N. Amapane, R. Arcidiacono, S. Argiro, M. Arneodo, N. Bartosik, R. Bellan, C. Biino, A. Cappati, N. Cartiglia, F. Cenna, S. Cometti, M. Costa, R. Covarelli, N. Demaria, B. Kiani, C. Mariotti, S. Maselli, E. Migliore, V. Monaco, E. Monteil, M. Monteno, M. M. Obertino, L. Pacher, N. Pastrone, M. Pelliccioni, G. L. Pinna Angioni, A. Romero, M. Ruspa, R. Sacchi, R. Salvatico, K. Shchelina, V. Sola, A. Solano, D. Soldi, A. Staiano, S. Belforte, V. Candelise, M. Casarsa, F. Cossutti, A. Da Rold, G. Della Ricca, F. Vazzoler, A. Zanetti, D. H. Kim, G. N. Kim, M. S. Kim, J. Lee, S. Lee, S. W. Lee, C. S. Moon, Y. D. Oh, S. I. Pak, S. Sekmen, D. C. Son, Y. C. Yang, H. Kim, D. H. Moon, G. Oh, B. Francois, J. Goh, T. J. Kim, S. Cho, S. Choi, Y. Go, D. Gyun, S. Ha, B. Hong, Y. Jo, K. Lee, K. S. Lee, S. Lee, J. Lim, S. K. Park, Y. Roh, H. S. Kim, J. Almond, J. Kim, J. S. Kim, H. Lee, K. Lee, K. Nam, S. B. Oh, B. C. Radburn-Smith, S. h. Seo, U. K. Yang, H. D. Yoo, G. B. Yu, D. Jeon, H. Kim, J. H. Kim, J. S. H. Lee, I. C. Park, Y. Choi, C. Hwang, J. Lee, I. Yu, V. Dudenas, A. Juodagalvis, J. Vaitkus, Z. A. Ibrahim, M. A. B. Md Ali, F. Mohamad Idris, W. A. T. Wan Abdullah, M. N. Yusli, Z. Zolkapli, J. F. Benitez, A. Castaneda Hernandez, J. A. Murillo Quijada, H. Castilla-Valdez, E. De La Cruz-Burelo, M. C. Duran-Osuna, I. Heredia-De La Cruz, R. Lopez-Fernandez, J. Mejia Guisao, R. I. Rabadan-Trejo, M. Ramirez-Garcia, G. Ramirez-Sanchez, R. Reyes-Almanza, A. Sanchez-Hernandez, S. Carrillo Moreno, C. Oropeza Barrera, F. Vazquez Valencia, J. Eysermans, I. Pedraza, H. A. Salazar Ibarguen, C. Uribe Estrada, A. Morelos Pineda, D. Krofcheck, S. Bheesette, P. H. Butler, A. Ahmad, M. Ahmad, M. I. Asghar, Q. Hassan, H. R. Hoorani, W. A. Khan, M. A. Shah, M. Shoaib, M. Waqas, H. Bialkowska, M. Bluj, B. Boimska, T. Frueboes, M. Górski, M. Kazana, M. Szleper, P. Traczyk, P. Zalewski, K. Bunkowski, A. Byszuk, K. Doroba, A. Kalinowski, M. Konecki, J. Krolikowski, M. Misiura, M. Olszewski, A. Pyskir, M. Walczak, M. Araujo, P. Bargassa, C. Beirão Da CruzE Silva, A. Di Francesco, P. Faccioli, B. Galinhas, M. Gallinaro, J. Hollar, N. Leonardo, J. Seixas, G. Strong, O. Toldaiev, J. Varela, S. Afanasiev, P. Bunin, M. Gavrilenko, I. Golutvin, I. Gorbunov, A. Kamenev, V. Karjavine, A. Lanev, A. Malakhov, V. Matveev, P. Moisenz, V. Palichik, V. Perelygin, S. Shmatov, S. Shulha, N. Skatchkov, V. Smirnov, N. Voytishin, A. Zarubin, V. Golovtsov, Y. Ivanov, V. Kim, E. Kuznetsova, P. Levchenko, V. Murzin, V. Oreshkin, I. Smirnov, D. Sosnov, V. Sulimov, L. Uvarov, S. Vavilov, A. Vorobyev, Yu. Andreev, A. Dermenev, S. Gninenko, N. Golubev, A. Karneyeu, M. Kirsanov, N. Krasnikov, A. Pashenkov, A. Shabanov, D. Tlisov, A. Toropin, V. Epshteyn, V. Gavrilov, N. Lychkovskaya, V. Popov, I. Pozdnyakov, G. Safronov, A. Spiridonov, A. Stepennov, V. Stolin, M. Toms, E. Vlasov, A. Zhokin, T. Aushev, M. Chadeeva, D. Philippov, E. Popova, V. Rusinov, V. Andreev, M. Azarkin, I. Dremin, M. Kirakosyan, A. Terkulov, A. Belyaev, E. Boos, M. Dubinin, L. Dudko, A. Ershov, A. Gribushin, V. Klyukhin, O. Kodolova, I. Lokhtin, S. Obraztsov, S. Petrushanko, V. Savrin, A. Snigirev, A. Barnyakov, V. Blinov, T. Dimova, L. Kardapoltsev, Y. Skovpen, I. Azhgirey, I. Bayshev, S. Bitioukov, V. Kachanov, A. Kalinin, D. Konstantinov, P. Mandrik, V. Petrov, R. Ryutin, S. Slabospitskii, A. Sobol, S. Troshin, N. Tyurin, A. Uzunian, A. Volkov, A. Babaev, S. Baidali, V. Okhotnikov, P. Adzic, P. Cirkovic, D. Devetak, M. Dordevic, J. Milosevic, J. Alcaraz Maestre, A. Álvarez Fernández, I. Bachiller, M. Barrio Luna, J. A. Brochero Cifuentes, M. Cerrada, N. Colino, B. De La Cruz, A. Delgado Peris, C. Fernandez Bedoya, J. P. Fernández Ramos, J. Flix, M. C. Fouz, O. Gonzalez Lopez, S. Goy Lopez, J. M. Hernandez, M. I. Josa, D. Moran, A. Pérez-Calero Yzquierdo, J. Puerta Pelayo, I. Redondo, L. Romero, S. Sánchez Navas, M. S. Soares, A. Triossi, C. Albajar, J. F. de Trocóniz, J. Cuevas, C. Erice, J. Fernandez Menendez, S. Folgueras, I. Gonzalez Caballero, J. R. González Fernández, E. Palencia Cortezon, V. Rodríguez Bouza, S. Sanchez Cruz, J. M. Vizan Garcia, I. J. Cabrillo, A. Calderon, B. Chazin Quero, J. Duarte Campderros, M. Fernandez, P. J. Fernández Manteca, A. García Alonso, J. Garcia-Ferrero, G. Gomez, A. Lopez Virto, J. Marco, C. Martinez Rivero, P. Martinez Ruiz del Arbol, F. Matorras, J. Piedra Gomez, C. Prieels, T. Rodrigo, A. Ruiz-Jimeno, L. Scodellaro, N. Trevisani, I. Vila, R. Vilar Cortabitarte, N. Wickramage, D. Abbaneo, B. Akgun, E. Auffray, G. Auzinger, P. Baillon, A. H. Ball, D. Barney, J. Bendavid, M. Bianco, A. Bocci, C. Botta, E. Brondolin, T. Camporesi, M. Cepeda, G. Cerminara, E. Chapon, Y. Chen, G. Cucciati, D. d’Enterria, A. Dabrowski, N. Daci, V. Daponte, A. David, A. De Roeck, N. Deelen, M. Dobson, M. Dünser, N. Dupont, A. Elliott-Peisert, P. Everaerts, F. Fallavollita, D. Fasanella, G. Franzoni, J. Fulcher, W. Funk, D. Gigi, A. Gilbert, K. Gill, F. Glege, M. Gruchala, M. Guilbaud, D. Gulhan, J. Hegeman, C. Heidegger, V. Innocente, A. Jafari, P. Janot, O. Karacheban, J. Kieseler, A. Kornmayer, M. Krammer, C. Lange, P. Lecoq, C. Lourenço, L. Malgeri, M. Mannelli, A. Massironi, F. Meijers, J. A. Merlin, S. Mersi, E. Meschi, P. Milenovic, F. Moortgat, M. Mulders, J. Ngadiuba, S. Nourbakhsh, S. Orfanelli, L. Orsini, F. Pantaleo, L. Pape, E. Perez, M. Peruzzi, A. Petrilli, G. Petrucciani, A. Pfeiffer, M. Pierini, F. M. Pitters, D. Rabady, A. Racz, T. Reis, M. Rovere, H. Sakulin, C. Schäfer, C. Schwick, M. Selvaggi, A. Sharma, P. Silva, P. Sphicas, A. Stakia, J. Steggemann, D. Treille, A. Tsirou, A. Vartak, V. Veckalns, M. Verzetti, W. D. Zeuner, L. Caminada, K. Deiters, W. Erdmann, R. Horisberger, Q. Ingram, H. C. Kaestli, D. Kotlinski, U. Langenegger, T. Rohe, S. A. Wiederkehr, M. Backhaus, L. Bäni, P. Berger, N. Chernyavskaya, G. Dissertori, M. Dittmar, M. Donegà, C. Dorfer, T. A. Gómez Espinosa, C. Grab, D. Hits, T. Klijnsma, W. Lustermann, R. A. Manzoni, M. Marionneau, M. T. Meinhard, F. Micheli, P. Musella, F. Nessi-Tedaldi, F. Pauss, G. Perrin, L. Perrozzi, S. Pigazzini, C. Reissel, D. Ruini, D. A. Sanz Becerra, M. Schönenberger, L. Shchutska, V. R. Tavolaro, K. Theofilatos, M. L. Vesterbacka Olsson, R. Wallny, D. H. Zhu, T. K. Aarrestad, C. Amsler, D. Brzhechko, M. F. Canelli, A. De Cosa, R. Del Burgo, S. Donato, C. Galloni, T. Hreus, B. Kilminster, S. Leontsinis, I. Neutelings, G. Rauco, P. Robmann, D. Salerno, K. Schweiger, C. Seitz, Y. Takahashi, A. Zucchetta, T. H. Doan, R. Khurana, C. M. Kuo, W. Lin, A. Pozdnyakov, S. S. Yu, P. Chang, Y. Chao, K. F. Chen, P. H. Chen, W.-S. Hou, Y. F. Liu, R.-S. Lu, E. Paganis, A. Psallidas, A. Steen, B. Asavapibhop, N. Srimanobhas, N. Suwonjandee, M. N. Bakirci, A. Bat, F. Boran, S. Damarseckin, Z. S. Demiroglu, F. Dolek, C. Dozen, I. Dumanoglu, G. Gokbulut, Y. Guler, E. Gurpinar, I. Hos, C. Isik, E. E. Kangal, O. Kara, A. Kayis Topaksu, U. Kiminsu, M. Oglakci, G. Onengut, K. Ozdemir, S. Ozturk, B. Tali, U. G. Tok, H. Topakli, S. Turkcapar, I. S. Zorbakir, C. Zorbilmez, B. Isildak, G. Karapinar, M. Yalvac, M. Zeyrek, I. O. Atakisi, E. Gülmez, M. Kaya, O. Kaya, S. Ozkorucuklu, S. Tekten, E. A. Yetkin, M. N. Agaras, A. Cakir, K. Cankocak, Y. Komurcu, S. Sen, B. Grynyov, L. Levchuk, F. Ball, J. J. Brooke, D. Burns, E. Clement, D. Cussans, O. Davignon, H. Flacher, J. Goldstein, G. P. Heath, H. F. Heath, L. Kreczko, D. M. Newbold, S. Paramesvaran, B. Penning, T. Sakuma, D. Smith, V. J. Smith, J. Taylor, A. Titterton, K. W. Bell, A. Belyaev, C. Brew, R. M. Brown, D. Cieri, D. J. A. Cockerill, J. A. Coughlan, K. Harder, S. Harper, J. Linacre, K. Manolopoulos, E. Olaiya, D. Petyt, C. H. Shepherd-Themistocleous, A. Thea, I. R. Tomalin, T. Williams, W. J. Womersley, R. Bainbridge, P. Bloch, J. Borg, S. Breeze, O. Buchmuller, A. Bundock, D. Colling, P. Dauncey, G. Davies, M. Della Negra, R. Di Maria, G. Hall, G. Iles, T. James, M. Komm, C. Laner, L. Lyons, A.-M. Magnan, S. Malik, A. Martelli, J. Nash, A. Nikitenko, V. Palladino, M. Pesaresi, D. M. Raymond, A. Richards, A. Rose, E. Scott, C. Seez, A. Shtipliyski, G. Singh, M. Stoye, T. Strebler, S. Summers, A. Tapper, K. Uchida, T. Virdee, N. Wardle, D. Winterbottom, J. Wright, S. C. Zenz, J. E. Cole, P. R. Hobson, A. Khan, P. Kyberd, C. K. Mackay, A. Morton, I. D. Reid, L. Teodorescu, S. Zahid, K. Call, J. Dittmann, K. Hatakeyama, H. Liu, C. Madrid, B. McMaster, N. Pastika, C. Smith, R. Bartek, A. Dominguez, A. Buccilli, S. I. Cooper, C. Henderson, P. Rumerio, C. West, D. Arcaro, T. Bose, D. Gastler, S. Girgis, D. Pinna, C. Richardson, J. Rohlf, L. Sulak, D. Zou, G. Benelli, B. Burkle, X. Coubez, D. Cutts, M. Hadley, J. Hakala, U. Heintz, J. M. Hogan, K. H. M. Kwok, E. Laird, G. Landsberg, J. Lee, Z. Mao, M. Narain, S. Sagir, R. Syarif, E. Usai, D. Yu, R. Band, C. Brainerd, R. Breedon, D. Burns, M. Calderon De La Barca Sanchez, M. Chertok, J. Conway, R. Conway, P. T. Cox, R. Erbacher, C. Flores, G. Funk, W. Ko, O. Kukral, R. Lander, M. Mulhearn, D. Pellett, J. Pilot, S. Shalhout, M. Shi, D. Stolp, D. Taylor, K. Tos, M. Tripathi, Z. Wang, F. Zhang, M. Bachtis, C. Bravo, R. Cousins, A. Dasgupta, S Erhan, A. Florent, J. Hauser, M. Ignatenko, N. Mccoll, S. Regnard, D. Saltzberg, C. Schnaible, V. Valuev, E. Bouvier, K. Burt, R. Clare, J. W. Gary, S. M. A. Ghiasi Shirazi, G. Hanson, G. Karapostoli, E. Kennedy, F. Lacroix, O. R. Long, M. Olmedo Negrete, M. I. Paneva, W. Si, L. Wang, H. Wei, S. Wimpenny, B. R. Yates, J. G. Branson, P. Chang, S. Cittolin, M. Derdzinski, R. Gerosa, D. Gilbert, B. Hashemi, A. Holzner, D. Klein, G. Kole, V. Krutelyov, J. Letts, M. Masciovecchio, S. May, D. Olivito, S. Padhi, M. Pieri, V. Sharma, M. Tadel, J. Wood, F. Würthwein, A. Yagil, G. Zevi Della Porta, N. Amin, R. Bhandari, C. Campagnari, M. Citron, V. Dutta, M. Franco Sevilla, L. Gouskos, R. Heller, J. Incandela, H. Mei, A. Ovcharova, H. Qu, J. Richman, D. Stuart, I. Suarez, S. Wang, J. Yoo, D. Anderson, A. Bornheim, J. M. Lawhorn, N. Lu, H. B. Newman, T. Q. Nguyen, J. Pata, M. Spiropulu, J. R. Vlimant, R. Wilkinson, S. Xie, Z. Zhang, R. Y. Zhu, M. B. Andrews, T. Ferguson, T. Mudholkar, M. Paulini, M. Sun, I. Vorobiev, M. Weinberg, J. P. Cumalat, W. T. Ford, F. Jensen, A. Johnson, E. MacDonald, T. Mulholland, R. Patel, A. Perloff, K. Stenson, K. A. Ulmer, S. R. Wagner, J. Alexander, J. Chaves, Y. Cheng, J. Chu, A. Datta, K. Mcdermott, N. Mirman, J. R. Patterson, D. Quach, A. Rinkevicius, A. Ryd, L. Skinnari, L. Soffi, S. M. Tan, Z. Tao, J. Thom, J. Tucker, P. Wittich, M. Zientek, S. Abdullin, M. Albrow, M. Alyari, G. Apollinari, A. Apresyan, A. Apyan, S. Banerjee, L. A. T. Bauerdick, A. Beretvas, J. Berryhill, P. C. Bhat, K. Burkett, J. N. Butler, A. Canepa, G. B. Cerati, H. W. K. Cheung, F. Chlebana, M. Cremonesi, J. Duarte, V. D. Elvira, J. Freeman, Z. Gecse, E. Gottschalk, L. Gray, D. Green, S. Grünendahl, O. Gutsche, J. Hanlon, R. M. Harris, S. Hasegawa, J. Hirschauer, Z. Hu, B. Jayatilaka, S. Jindariani, M. Johnson, U. Joshi, B. Klima, M. J. Kortelainen, B. Kreis, S. Lammel, D. Lincoln, R. Lipton, M. Liu, T. Liu, J. Lykken, K. Maeshima, J. M. Marraffino, D. Mason, P. McBride, P. Merkel, S. Mrenna, S. Nahn, V. O’Dell, K. Pedro, C. Pena, O. Prokofyev, G. Rakness, F. Ravera, A. Reinsvold, L. Ristori, A. Savoy-Navarro, B. Schneider, E. Sexton-Kennedy, A. Soha, W. J. Spalding, L. Spiegel, S. Stoynev, J. Strait, N. Strobbe, L. Taylor, S. Tkaczyk, N. V. Tran, L. Uplegger, E. W. Vaandering, C. Vernieri, M. Verzocchi, R. Vidal, M. Wang, H. A. Weber, A. Whitbeck, D. Acosta, P. Avery, P. Bortignon, D. Bourilkov, A. Brinkerhoff, L. Cadamuro, A. Carnes, D. Curry, R. D. Field, S. V. Gleyzer, B. M. Joshi, J. Konigsberg, A. Korytov, K. H. Lo, P. Ma, K. Matchev, G. Mitselmakher, D. Rosenzweig, K. Shi, D. Sperka, J. Wang, S. Wang, X. Zuo, Y. R. Joshi, S. Linn, A. Ackert, T. Adams, A. Askew, S. Hagopian, V. Hagopian, K. F. Johnson, T. Kolberg, G. Martinez, T. Perry, H. Prosper, A. Saha, C. Schiber, R. Yohay, M. M. Baarmand, V. Bhopatkar, S. Colafranceschi, M. Hohlmann, D. Noonan, M. Rahmani, T. Roy, M. Saunders, F. Yumiceva, M. R. Adams, L. Apanasevich, D. Berry, R. R. Betts, R. Cavanaugh, X. Chen, S. Dittmer, O. Evdokimov, C. E. Gerber, D. A. Hangal, D. J. Hofman, K. Jung, J. Kamin, C. Mills, M. B. Tonjes, N. Varelas, H. Wang, X. Wang, Z. Wu, J. Zhang, M. Alhusseini, B. Bilki, W. Clarida, K. Dilsiz, S. Durgut, R. P. Gandrajula, M. Haytmyradov, V. Khristenko, J.-P. Merlo, A. Mestvirishvili, A. Moeller, J. Nachtman, H. Ogul, Y. Onel, F. Ozok, A. Penzo, C. Snyder, E. Tiras, J. Wetzel, B. Blumenfeld, A. Cocoros, N. Eminizer, D. Fehling, L. Feng, A. V. Gritsan, W. T. Hung, P. Maksimovic, J. Roskes, U. Sarica, M. Swartz, M. Xiao, A. Al-bataineh, P. Baringer, A. Bean, S. Boren, J. Bowen, A. Bylinkin, J. Castle, S. Khalil, A. Kropivnitskaya, D. Majumder, W. Mcbrayer, M. Murray, C. Rogan, S. Sanders, E. Schmitz, J. D. Tapia Takaki, Q. Wang, S. Duric, A. Ivanov, K. Kaadze, D. Kim, Y. Maravin, D. R. Mendis, T. Mitchell, A. Modak, A. Mohammadi, F. Rebassoo, D. Wright, A. Baden, O. Baron, A. Belloni, S. C. Eno, Y. Feng, C. Ferraioli, N. J. Hadley, S. Jabeen, G. Y. Jeng, R. G. Kellogg, J. Kunkle, A. C. Mignerey, S. Nabili, F. Ricci-Tam, M. Seidel, Y. H. Shin, A. Skuja, S. C. Tonwar, K. Wong, D. Abercrombie, B. Allen, V. Azzolini, A. Baty, G. Bauer, R. Bi, S. Brandt, W. Busza, I. A. Cali, M. D’Alfonso, Z. Demiragli, G. Gomez Ceballos, M. Goncharov, P. Harris, D. Hsu, M. Hu, Y. Iiyama, G. M. Innocenti, M. Klute, D. Kovalskyi, Y.-J. Lee, P. D. Luckey, B. Maier, A. C. Marini, C. Mcginn, C. Mironov, S. Narayanan, X. Niu, C. Paus, D. Rankin, C. Roland, G. Roland, Z. Shi, G. S. F. Stephans, K. Sumorok, K. Tatar, D. Velicanu, J. Wang, T. W. Wang, B. Wyslouch, A. C. Benvenuti, R. M. Chatterjee, A. Evans, P. Hansen, J. Hiltbrand, Sh. Jain, S. Kalafut, M. Krohn, Y. Kubota, Z. Lesko, J. Mans, R. Rusack, M. A. Wadud, J. G. Acosta, S. Oliveros, E. Avdeeva, K. Bloom, D. R. Claes, C. Fangmeier, F. Golf, R. Gonzalez Suarez, R. Kamalieddin, I. Kravchenko, J. Monroy, J. E. Siado, G. R. Snow, B. Stieger, A. Godshalk, C. Harrington, I. Iashvili, A. Kharchilava, C. Mclean, D. Nguyen, A. Parker, S. Rappoccio, B. Roozbahani, G. Alverson, E. Barberis, C. Freer, Y. Haddad, A. Hortiangtham, G. Madigan, D. M. Morse, T. Orimoto, A. Tishelman-charny, T. Wamorkar, B. Wang, A. Wisecarver, D. Wood, S. Bhattacharya, J. Bueghly, O. Charaf, T. Gunter, K. A. Hahn, N. Odell, M. H. Schmitt, K. Sung, M. Trovato, M. Velasco, R. Bucci, N. Dev, M. Hildreth, K. Hurtado Anampa, C. Jessop, D. J. Karmgard, K. Lannon, W. Li, N. Loukas, N. Marinelli, F. Meng, C. Mueller, Y. Musienko, M. Planer, R. Ruchti, P. Siddireddy, G. Smith, S. Taroni, M. Wayne, A. Wightman, M. Wolf, A. Woodard, J. Alimena, L. Antonelli, B. Bylsma, L. S. Durkin, S. Flowers, B. Francis, C. Hill, W. Ji, T. Y. Ling, W. Luo, B. L. Winer, S. Cooperstein, P. Elmer, J. Hardenbrook, N. Haubrich, S. Higginbotham, A. Kalogeropoulos, S. Kwan, D. Lange, M. T. Lucchini, J. Luo, D. Marlow, K. Mei, I. Ojalvo, J. Olsen, C. Palmer, P. Piroué, J. Salfeld-Nebgen, D. Stickland, C. Tully, S. Malik, S. Norberg, A. Barker, V. E. Barnes, S. Das, L. Gutay, M. Jones, A. W. Jung, A. Khatiwada, B. Mahakud, D. H. Miller, N. Neumeister, C. C. Peng, S. Piperov, H. Qiu, J. F. Schulte, J. Sun, F. Wang, R. Xiao, W. Xie, T. Cheng, J. Dolen, N. Parashar, Z. Chen, K. M. Ecklund, S. Freed, F. J. M. Geurts, M. Kilpatrick, Arun Kumar, W. Li, B. P. Padley, R. Redjimi, J. Roberts, J. Rorie, W. Shi, Z. Tu, A. Zhang, A. Bodek, P. de Barbaro, R. Demina, Y. t. Duh, J. L. Dulemba, C. Fallon, T. Ferbel, M. Galanti, A. Garcia-Bellido, J. Han, O. Hindrichs, A. Khukhunaishvili, E. Ranken, P. Tan, R. Taus, B. Chiarito, J. P. Chou, Y. Gershtein, E. Halkiadakis, A. Hart, M. Heindl, E. Hughes, S. Kaplan, R. Kunnawalkam Elayavalli, S. Kyriacou, I. Laflotte, A. Lath, R. Montalvo, K. Nash, M. Osherson, H. Saka, S. Salur, S. Schnetzer, D. Sheffield, S. Somalwar, R. Stone, S. Thomas, P. Thomassen, A. G. Delannoy, J. Heideman, G. Riley, S. Spanier, O. Bouhali, A. Celik, M. Dalchenko, M. De Mattia, A. Delgado, S. Dildick, R. Eusebi, J. Gilmore, T. Huang, T. Kamon, S. Luo, D. Marley, R. Mueller, D. Overton, L. Perniè, D. Rathjens, A. Safonov, N. Akchurin, J. Damgov, F. De Guio, P. R. Dudero, S. Kunori, K. Lamichhane, S. W. Lee, T. Mengke, S. Muthumuni, T. Peltola, S. Undleeb, I. Volobouev, Z. Wang, S. Greene, A. Gurrola, R. Janjam, W. Johns, C. Maguire, A. Melo, H. Ni, K. Padeken, F. Romeo, J. D. Ruiz Alvarez, P. Sheldon, S. Tuo, J. Velkovska, M. Verweij, Q. Xu, M. W. Arenton, P. Barria, B. Cox, R. Hirosky, M. Joyce, A. Ledovskoy, H. Li, C. Neu, T. Sinthuprasith, Y. Wang, E. Wolfe, F. Xia, R. Harr, P. E. Karchin, N. Poudyal, J. Sturdy, P. Thapa, S. Zaleski, J. Buchanan, C. Caillol, D. Carlsmith, S. Dasu, I. De Bruyn, L. Dodd, B. Gomber, M. Grothe, M. Herndon, A. Hervé, U. Hussain, P. Klabbers, A. Lanaro, K. Long, R. Loveless, T. Ruggles, A. Savin, V. Sharma, N. Smith, W. H. Smith, N. Woods, CMS Collaboration

**Affiliations:** 1 Yerevan Physics Institute, Yerevan, Armenia; 2 Institut für Hochenergiephysik, Wien, Austria; 3 Institute for Nuclear Problems, Minsk, Belarus; 4 Universiteit Antwerpen, Antwerpen, Belgium; 5 Vrije Universiteit Brussel, Brussels, Belgium; 6 Université Libre de Bruxelles, Brussels, Belgium; 7 Ghent University, Ghent, Belgium; 8 Université Catholique de Louvain, Louvain-la-Neuve, Belgium; 9 Centro Brasileiro de Pesquisas Fisicas, Rio de Janeiro, Brazil; 10 Universidade do Estado do Rio de Janeiro, Rio de Janeiro, Brazil; 11 Universidade Estadual Paulista , Universidade Federal do ABC, São Paulo, Brazil; 12 Institute for Nuclear Research and Nuclear Energy, Bulgarian Academy of Sciences, Sofia, Bulgaria; 13 University of Sofia, Sofia, Bulgaria; 14 Beihang University, Beijing, China; 15 Institute of High Energy Physics, Beijing, China; 16 State Key Laboratory of Nuclear Physics and Technology, Peking University, Beijing, China; 17 Tsinghua University, Beijing, China; 18 Universidad de Los Andes, Bogota, Colombia; 19 University of Split, Faculty of Electrical Engineering, Mechanical Engineering and Naval Architecture, Split, Croatia; 20 University of Split, Faculty of Science, Split, Croatia; 21 Institute Rudjer Boskovic, Zagreb, Croatia; 22 University of Cyprus, Nicosia, Cyprus; 23 Charles University, Prague, Czech Republic; 24 Escuela Politecnica Nacional, Quito, Ecuador; 25 Universidad San Francisco de Quito, Quito, Ecuador; 26 Academy of Scientific Research and Technology of the Arab Republic of Egypt, Egyptian Network of High Energy Physics, Cairo, Egypt; 27 National Institute of Chemical Physics and Biophysics, Tallinn, Estonia; 28 Department of Physics, University of Helsinki, Helsinki, Finland; 29 Helsinki Institute of Physics, Helsinki, Finland; 30 Lappeenranta University of Technology, Lappeenranta, Finland; 31 IRFU, CEA, Université Paris-Saclay, Gif-sur-Yvette, France; 32 Laboratoire Leprince-Ringuet, Ecole polytechnique, CNRS/IN2P3, Université Paris-Saclay, Palaiseau, France; 33 Université de Strasbourg, CNRS, IPHC UMR 7178, Strasbourg, France; 34 Centre de Calcul de l’Institut National de Physique Nucleaire et de Physique des Particules, CNRS/IN2P3, Villeurbanne, France; 35 Université de Lyon, Université Claude Bernard Lyon 1, CNRS-IN2P3, Institut de Physique Nucléaire de Lyon, Villeurbanne, France; 36 Georgian Technical University, Tbilisi, Georgia; 37 Tbilisi State University, Tbilisi, Georgia; 38 RWTH Aachen University, I. Physikalisches Institut, Aachen, Germany; 39 RWTH Aachen University, III. Physikalisches Institut A, Aachen, Germany; 40 RWTH Aachen University, III. Physikalisches Institut B, Aachen, Germany; 41 Deutsches Elektronen-Synchrotron, Hamburg, Germany; 42 University of Hamburg, Hamburg, Germany; 43 Karlsruher Institut fuer Technologie, Karlsruhe, Germany; 44 Institute of Nuclear and Particle Physics (INPP), NCSR Demokritos, Aghia Paraskevi, Greece; 45 National and Kapodistrian University of Athens, Athens, Greece; 46 National Technical University of Athens, Athens, Greece; 47 University of Ioánnina, Ioánnina, Greece; 48 MTA-ELTE Lendület CMS Particle and Nuclear Physics Group, Eötvös Loránd University, Budapest, Hungary; 49 Wigner Research Centre for Physics, Budapest, Hungary; 50 Institute of Nuclear Research ATOMKI, Debrecen, Hungary; 51 Institute of Physics, University of Debrecen, Debrecen, Hungary; 52 Indian Institute of Science (IISc), Bangalore, India; 53 National Institute of Science Education and Research, HBNI, Bhubaneswar, India; 54 Panjab University, Chandigarh, India; 55 University of Delhi, Delhi, India; 56 Saha Institute of Nuclear Physics, HBNI, Kolkata, India; 57 Indian Institute of Technology Madras, Madras, India; 58 Bhabha Atomic Research Centre, Mumbai, India; 59 Tata Institute of Fundamental Research-A, Mumbai, India; 60 Tata Institute of Fundamental Research-B, Mumbai, India; 61 Indian Institute of Science Education and Research (IISER), Pune, India; 62 Institute for Research in Fundamental Sciences (IPM), Tehran, Iran; 63 University College Dublin, Dublin, Ireland; 64 INFN Sezione di Bari, Università di Bari, Politecnico di Bari, Bari, Italy; 65 INFN Sezione di Bologna, Università di Bologna, Bologna, Italy; 66 INFN Sezione di Catania, Università di Catania, Catania, Italy; 67 INFN Sezione di Firenze, Università di Firenze, Florence, Italy; 68 INFN Laboratori Nazionali di Frascati, Frascati, Italy; 69 INFN Sezione di Genova, Università di Genova, Genoa, Italy; 70 INFN Sezione di Milano-Bicocca, Università di Milano-Bicocca, Milan, Italy; 71 INFN Sezione di Napoli, Università di Napoli ’Federico II’ , Napoli, Italy, Università della Basilicata, Potenza, Italy, Università G. Marconi, Rome, Italy; 72 INFN Sezione di Padova, Università di Padova, Padova, Italy, Università di Trento, Trento, Italy; 73 INFN Sezione di Pavia, Università di Pavia, Pavia, Italy; 74 INFN Sezione di Perugia, Università di Perugia, Perugia, Italy; 75 INFN Sezione di Pisa, Università di Pisa, Scuola Normale Superiore di Pisa, Pisa, Italy; 76 INFN Sezione di Roma, Sapienza Università di Roma, Rome, Italy; 77 INFN Sezione di Torino, Università di Torino, Torino, Italy, Università del Piemonte Orientale, Novara, Italy; 78 INFN Sezione di Trieste, Università di Trieste, Trieste, Italy; 79 Kyungpook National University, Daegu, Korea; 80 Chonnam National University, Institute for Universe and Elementary Particles, Kwangju, Korea; 81 Hanyang University, Seoul, Korea; 82 Korea University, Seoul, Korea; 83 Sejong University, Seoul, Korea; 84 Seoul National University, Seoul, Korea; 85 University of Seoul, Seoul, Korea; 86 Sungkyunkwan University, Suwon, Korea; 87 Vilnius University, Vilnius, Lithuania; 88 National Centre for Particle Physics, Universiti Malaya, Kuala Lumpur, Malaysia; 89 Universidad de Sonora (UNISON), Hermosillo, Mexico; 90 Centro de Investigacion y de Estudios Avanzados del IPN, Mexico City, Mexico; 91 Universidad Iberoamericana, Mexico City, Mexico; 92 Benemerita Universidad Autonoma de Puebla, Puebla, Mexico; 93 Universidad Autónoma de San Luis Potosí, San Luis Potosí, Mexico; 94 University of Auckland, Auckland, New Zealand; 95 University of Canterbury, Christchurch, New Zealand; 96 National Centre for Physics, Quaid-I-Azam University, Islamabad, Pakistan; 97 National Centre for Nuclear Research, Swierk, Poland; 98 Institute of Experimental Physics, Faculty of Physics, University of Warsaw, Warsaw, Poland; 99 Laboratório de Instrumentação e Física Experimental de Partículas, Lisboa, Portugal; 100 Joint Institute for Nuclear Research, Dubna, Russia; 101 Petersburg Nuclear Physics Institute, Gatchina (St. Petersburg), Russia; 102 Institute for Nuclear Research, Moscow, Russia; 103 Institute for Theoretical and Experimental Physics, Moscow, Russia; 104 Moscow Institute of Physics and Technology, Moscow, Russia; 105 National Research Nuclear University ’Moscow Engineering Physics Institute’ (MEPhI), Moscow, Russia; 106 P.N. Lebedev Physical Institute, Moscow, Russia; 107 Skobeltsyn Institute of Nuclear Physics, Lomonosov Moscow State University, Moscow, Russia; 108 Novosibirsk State University (NSU), Novosibirsk, Russia; 109 Institute for High Energy Physics of National Research Centre ’Kurchatov Institute’, Protvino, Russia; 110 National Research Tomsk Polytechnic University, Tomsk, Russia; 111 University of Belgrade, Faculty of Physics and Vinca Institute of Nuclear Sciences, Belgrade, Serbia; 112 Centro de Investigaciones Energéticas Medioambientales y Tecnológicas (CIEMAT), Madrid, Spain; 113 Universidad Autónoma de Madrid, Madrid, Spain; 114 Universidad de Oviedo, Oviedo, Spain; 115 Instituto de Física de Cantabria (IFCA), CSIC-Universidad de Cantabria, Santander, Spain; 116 Department of Physics, University of Ruhuna, Matara, Sri Lanka; 117 CERN, European Organization for Nuclear Research, Geneva, Switzerland; 118 Paul Scherrer Institut, Villigen, Switzerland; 119 ETH Zurich - Institute for Particle Physics and Astrophysics (IPA), Zurich, Switzerland; 120 Universität Zürich, Zurich, Switzerland; 121 National Central University, Chung-Li, Taiwan; 122 National Taiwan University (NTU), Taipei, Taiwan; 123 Faculty of Science, Department of Physics, Chulalongkorn University, Bangkok, Thailand; 124 Physics Department, Science and Art Faculty, Çukurova University, Adana, Turkey; 125 Physics Department, Middle East Technical University, Ankara, Turkey; 126 Bogazici University, Istanbul, Turkey; 127 Istanbul Technical University, Istanbul, Turkey; 128 Institute for Scintillation Materials of National Academy of Science of Ukraine, Kharkov, Ukraine; 129 National Scientific Center, Kharkov Institute of Physics and Technology, Kharkov, Ukraine; 130 University of Bristol, Bristol, UK; 131 Rutherford Appleton Laboratory, Didcot, UK; 132 Imperial College, London, UK; 133 Brunel University, Uxbridge, UK; 134 Baylor University, Waco, USA; 135 Catholic University of America, Washington, DC USA; 136 The University of Alabama, Tuscaloosa, USA; 137 Boston University, Boston, USA; 138 Brown University, Providence, USA; 139 University of California, Davis, Davis USA; 140 University of California, Los Angeles, USA; 141 University of California, Riverside, Riverside, USA; 142 University of California, San Diego, La Jolla, USA; 143 Department of Physics, University of California, Santa Barbara, Santa Barbara, USA; 144 California Institute of Technology, Pasadena, USA; 145 Carnegie Mellon University, Pittsburgh, USA; 146 University of Colorado Boulder, Boulder, USA; 147 Cornell University, Ithaca, USA; 148 Fermi National Accelerator Laboratory, Batavia, USA; 149 University of Florida, Gainesville, USA; 150 Florida International University, Miami, USA; 151 Florida State University, Tallahassee, USA; 152 Florida Institute of Technology, Melbourne, USA; 153 University of Illinois at Chicago (UIC), Chicago, USA; 154 The University of Iowa, Iowa City, USA; 155 Johns Hopkins University, Baltimore, USA; 156 The University of Kansas, Lawrence, USA; 157 Kansas State University, Manhattan, USA; 158 Lawrence Livermore National Laboratory, Livermore, USA; 159 University of Maryland, College Park, USA; 160 Massachusetts Institute of Technology, Cambridge, USA; 161 University of Minnesota, Minneapolis, USA; 162 University of Mississippi, Oxford, USA; 163 University of Nebraska-Lincoln, Lincoln, USA; 164 State University of New York at Buffalo, Buffalo, USA; 165 Northeastern University, Boston, USA; 166 Northwestern University, Evanston, USA; 167 University of Notre Dame, Notre Dame, USA; 168 The Ohio State University, Columbus, USA; 169 Princeton University, Princeton, USA; 170 University of Puerto Rico, Mayaguez, USA; 171 Purdue University, West Lafayette, USA; 172 Purdue University Northwest, Hammond, USA; 173 Rice University, Houston, USA; 174 University of Rochester, Rochester, USA; 175 Rutgers, The State University of New Jersey, Piscataway, USA; 176 University of Tennessee, Knoxville, USA; 177 Texas A & M University, College Station, USA; 178 Texas Tech University, Lubbock, USA; 179 Vanderbilt University, Nashville, USA; 180 University of Virginia, Charlottesville, USA; 181 Wayne State University, Detroit, USA; 182 University of Wisconsin - Madison, Madison, WI USA; 183 CERN, 1211 Geneva 23, Switzerland

## Abstract

A search for supersymmetry is presented based on events with at least one photon, jets, and large missing transverse momentum produced in proton–proton collisions at a center-of-mass energy of 13$\,\text {Te}\text {V}$. The data correspond to an integrated luminosity of 35.9$\,\text {fb}^{-1}$ and were recorded at the LHC with the CMS detector in 2016. The analysis characterizes signal-like events by categorizing the data into various signal regions based on the number of jets, the number of $\mathrm {b}$-tagged jets, and the missing transverse momentum. No significant excess of events is observed with respect to the expectations from standard model processes. Limits are placed on the gluino and top squark pair production cross sections using several simplified models of supersymmetric particle production with gauge-mediated supersymmetry breaking. Depending on the model and the mass of the next-to-lightest supersymmetric particle, the production of gluinos with masses as large as 2120$\,\text {Ge}\text {V}$ and the production of top squarks with masses as large as 1230$\,\text {Ge}\text {V}$ are excluded at 95% confidence level.

## Introduction

The standard model (SM) of particle physics successfully describes many phenomena, but lacks several necessary elements to provide a complete description of nature, including a source for the relic abundance of dark matter (DM) [1, 2] in the universe. In addition, the SM must resort to fine tuning [3–6] to explain the hierarchy between the Planck mass scale and the electroweak scale set by the vacuum expectation value of the Higgs field, the existence of which was recently confirmed by the observation of the Higgs boson ($\mathrm {H}$) [7, 8]. Supersymmetry (SUSY) [9–16] is an extension of the SM that can provide both a viable DM candidate and additional particles that inherently cancel large quantum corrections to the Higgs boson mass-squared term from the SM fields.

Supersymmetric models predict a bosonic superpartner for each SM fermion and a fermionic superpartner for each SM boson; each new particle’s spin differs from that of its SM partner by half a unit. SUSY also includes a second Higgs doublet. New colored states, such as gluinos ($\widetilde{\mathrm {g}}$) and top squarks ($\widetilde{\mathrm {t}}$), the superpartners of the gluon and the top quark, respectively, are expected to have masses on the order of 1$\,\text {Te}\text {V}$ to avoid fine tuning in the SM Higgs boson mass-squared term. In models that conserve *R*-parity [17], each superpartner carries a conserved quantum number that requires superpartners to be produced in pairs and causes the lightest SUSY particle (LSP) to be stable. The stable LSP can serve as a DM candidate.

The signatures targeted in this paper are motivated by models in which gauge-mediated SUSY breaking (GMSB) is responsible for separating the masses of the SUSY particles from those of their SM counterparts. In GMSB models, the gaugino masses are expected to be proportional to the size of their fundamental couplings. This includes the superpartner of the graviton, the gravitino ($\widetilde{\mathrm {G}}$), whose mass is proportional to $M_{\text {SB}}/M_{\text {Pl}}$, where $M_{\text {SB}}$ represents the scale of the SUSY breaking interactions and $M_{\text {Pl}}$ is the Planck scale where gravity is expected to become strong. GMSB permits a significantly lower symmetry-breaking scale than, e.g., gravity mediation, and therefore generically predicts that the $\widetilde{\mathrm {G}}$ is the LSP [18–20], with a mass often much less than 1$\,\text {Ge}\text {V}$. Correspondingly, the next-to-LSP (NLSP) is typically a neutralino, a superposition of the superpartners of the neutral bosons. The details of the quantum numbers of the NLSP play a large part in determining the phenomenology of GMSB models, including the relative frequencies of the Higgs bosons, $\mathrm {Z}$ bosons, and photons produced in the NLSP decay.

**Fig. 1:**
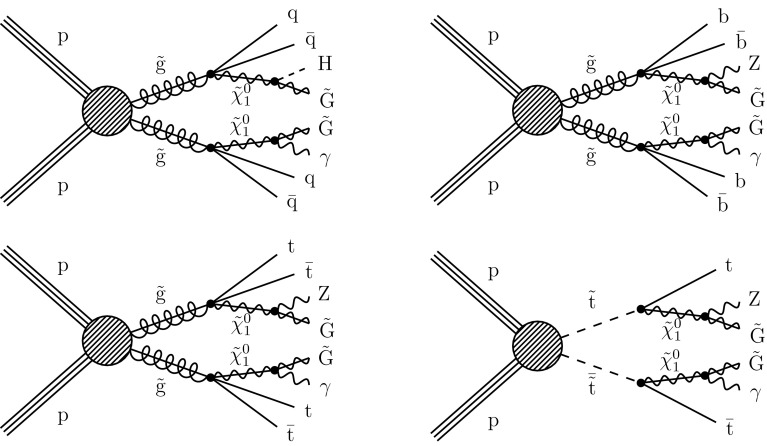
Example diagrams depicting the simplified models used, which are defined in the text. The top left diagram depicts the T5qqqqHG model, the top right diagram depicts the T5bbbbZG model, the bottom left diagram depicts the T5ttttZG model, and the bottom right depicts the T6ttZG model

The scenario of a natural SUSY spectrum with GMSB and *R*-parity conservation typically manifests as events with multiple jets, at least one photon, and large $p_{\mathrm {T}} ^\text {miss}$, the magnitude of the missing transverse momentum. Depending on the topology, these jets can arise from either light-flavored quarks ($\mathrm {u}$, $\mathrm {d}$, $\mathrm {s}$, $\mathrm {c}$) or $\mathrm {b}$ quarks. We study four simplified models [21–25]; example diagrams depicting these models are shown in Fig. 1. Three models involve gluino pair production (prefixed with T5), and one model involves top squark pair production (prefixed with T6). In the T5qqqqHG model, each gluino decays to a pair of light-flavored quarks ($\mathrm {q} \overline{\mathrm {q}}$) and a neutralino ($\widetilde{\chi }^{0}_{1}$). The T5bbbbZG and T5ttttZG models are similar to T5qqqqHG, except that the each pair of light-flavored quarks is replaced by a pair of bottom quarks ($\mathrm {b} \overline{\mathrm {b}}$) or a pair of top quarks (${\mathrm {t}\overline{\mathrm {t}}}$), respectively. In the T5qqqqHG model, the $\widetilde{\chi }^{0}_{1}$ decays either to an SM Higgs boson and a $\widetilde{\mathrm {G}}$ or to a photon and a $\widetilde{\mathrm {G}}$. The $\widetilde{\chi }^{0}_{1} \rightarrow \mathrm {H} \widetilde{\mathrm {G}}$ branching fraction is assumed to be 50%, and the smallest $\widetilde{\chi }^{0}_{1}$ mass considered is 127$\,\text {Ge}\text {V}$. In the T5bbbbZG and T5ttttZG models, the neutralinos decay to $\mathrm {Z} \widetilde{\mathrm {G}}$ and $\gamma \widetilde{\mathrm {G}}$ with equal probability. The T6ttZG model considers top squark pair production, with each top squark decaying into a top quark and a neutralino. The neutralino can then decay with equal probability to a photon and a $\widetilde{\mathrm {G}}$ or to a $\mathrm {Z}$ boson and a $\widetilde{\mathrm {G}}$. For the models involving the decay $\widetilde{\chi }^{0}_{1} \rightarrow \mathrm {Z} \widetilde{\mathrm {G}}$, we probe $\widetilde{\chi }^{0}_{1}$ masses down to 10$\,\text {Ge}\text {V}$. All decays of SUSY particles are assumed to be prompt. In all models, the mass $m_{\widetilde{\mathrm {G}}}$ is fixed to be 1$\,\text {Ge}\text {V}$, to be consistent with other published results. For the parameter space explored here, the kinematic properties do not depend strongly on the exact value of $m_{\widetilde{\mathrm {G}}}$.

The proton–proton ($\mathrm {p}\mathrm {p}$) collision data used in this search correspond to an integrated luminosity of 35.9$\,\text {fb}^{-1}$ and were collected with the CMS detector during the 2016 run of the CERN LHC [26]. Signal-like events with at least one photon are classified into signal regions depending on the number of jets $N_{\text {jets}}$, the number of tagged bottom quark jets $N_{{\mathrm {b}}\text {-jets}}$, and the $p_{\mathrm {T}} ^\text {miss}$. The expected yields from SM backgrounds are estimated using a combination of simulation and data control regions. We search for gluino or top squark pair production as an excess of observed data events compared to the expected background yields.

Previous searches for *R*-parity conserving SUSY with photons in the final state performed by the CMS Collaboration are documented in Refs. [27, 28]. Similar searches have also been performed by the ATLAS Collaboration [29–31]. This work improves on the previous results by identifying jets from $\mathrm {b}$ quarks, which can be produced by all of the signal models shown in Fig. 1. We also include additional signal regions that exploit high jet multiplicities for sensitivity to high-mass gluino models, and we rely more on observed data for the background estimations. These improvements enable us to explore targeted signal models that produce $\mathrm {b}$ quarks in the final state and are expected to improve sensitivity to the models explored in Refs. [27–31].

In this paper, a description of the CMS detector and simulation used are presented in Sect. 2. The event reconstruction and signal region selections are presented in Sect. 3. The methods used for predicting the SM backgrounds are presented in Sect. 4. Results are given in Sect. 5. The analysis is summarized in Sect. 6.

## Detector and simulation

A detailed description of the CMS detector, along with a definition of the coordinate system and pertinent kinematic variables, is given in Ref. [32]. Briefly, a cylindrical superconducting solenoid with an inner diameter of 6$\text { m}$ provides a 3.8$\text { T}$ axial magnetic field. Within the cylindrical volume are a silicon pixel and strip tracker, a lead tungstate crystal electromagnetic calorimeter (ECAL), and a brass and scintillator hadron calorimeter (HCAL). The tracking detectors cover the pseudorapidity range ${|\eta |<2.5}$. The ECAL and HCAL, each composed of a barrel and two endcap sections, cover ${|\eta |<3.0}$. Forward calorimeters extend the coverage to ${3.0<|\eta |<5.0}$. Muons are detected within ${|\eta |<2.4}$ by gas-ionization detectors embedded in the steel flux-return yoke outside the solenoid. The detector is nearly hermetic, permitting accurate measurements of $p_{\mathrm {T}} ^\text {miss}$. The CMS trigger is described in Ref. [33].

Monte Carlo (MC) simulation is used to design the analysis, to provide input for background estimation methods that use data control regions, and to predict event rates from simplified models. Simulated SM background processes include jets produced through the strong interaction, referred to as quantum chromodynamics (QCD) multijets, ${\mathrm {t}\overline{\mathrm {t}}}$+jets, $\mathrm {W}$+jets, $\mathrm {Z}$+jets, ${\gamma }\text {+jets}$, ${\mathrm {t}\overline{\mathrm {t}}} {\gamma }$, ${\mathrm {t}}{\gamma }$, and $\mathrm {V}$ ${\gamma }\text {+jets}$ ($\mathrm {V}$ = $\mathrm {Z}$, $\mathrm {W}$). The SM background events are generated using the MadGraph 5_amc@nlo v2.2.2 or v2.3.3 generator [34–36] at leading order (LO) in perturbative QCD, except ${\mathrm {t}\overline{\mathrm {t}}} {\gamma }$ and ${\mathrm {t}}{\gamma }$, which are generated at next-to-leading order (NLO). The cross sections used for normalization are computed at NLO or next-to-NLO [34, 37–39]. The QCD multijets, diboson ($\mathrm {V}$ $\gamma$), top quark, and vector boson plus jets events are generated with up to two, two, three, and four additional partons in the matrix element calculations, respectively. Any duplication of events between pairs of related processes – QCD multijets and ${\gamma }\text {+jets}$; ${\mathrm {t}\overline{\mathrm {t}}}$+jets and ${\mathrm {t}\overline{\mathrm {t}}} {\gamma }$; $\mathrm {W}$+jets and $\mathrm {W}$ ${\gamma }\text {+jets}$– is removed using generator information.

The NNPDF3.0 [40] LO (NLO) parton distribution functions (PDFs) are used for samples simulated at LO (NLO). Parton showering and hadronization are described using the pythia 8.212 generator [41] with the CUETP8M1 underlying event tune [42]. Partons generated with MadGraph 5_amc@nlo and pythia that would otherwise be counted twice are removed using the MLM [43] and FxFx [44] matching schemes in LO and NLO samples, respectively.

Signal samples are simulated at LO using the MadGraph 5_amc@nlo v2.3.3 generator and their yields are normalized using NLO plus next-to-leading logarithmic (NLL) cross sections [45–49]. The decays of gluinos, top squarks, and neutralinos are modeled with pythia.

The detector response to particles produced in the simulated collisions is modeled with the Geant4  [50] detector simulation package for SM processes. Because of the large number of SUSY signals considered, with various gluino, squark, and neutralino masses, the detector response for these processes is simulated with the CMS fast simulation [51, 52]. The results from the fast simulation generally agree with the results from the full simulation. Where there is disagreement, corrections are applied, most notably a correction of up to 10% to adjust for differences in the modeling of $p_{\mathrm {T}} ^\text {miss}$.

## Event reconstruction and selection

The CMS particle-flow (PF) algorithm [53] aims to reconstruct every particle in each event, using an optimal combination of information from all detector systems. Particle candidates are identified as charged hadrons, neutral hadrons, electrons, photons, or muons. For electron and photon PF candidates, further requirements are applied to the ECAL shower shape and the ratio of associated energies in the ECAL and HCAL [54, 55]. Similarly, for muon PF candidates, further requirements are applied to the matching between track segments in the silicon tracker and the muon detectors [56]. These further requirements improve the quality of the reconstruction. Electron and muon candidates are restricted to $|\eta |<2.5$ and $<2.4$, respectively. The $p_{\mathrm {T}} ^\text {miss}$ is calculated as the magnitude of the negative vector $p_{\mathrm {T}}$ sum of all PF candidates.

After all interaction vertices are reconstructed, the primary $\mathrm {p}\mathrm {p}$ interaction vertex is selected as the vertex with the largest $p_{\mathrm {T}} ^2$ sum of all physics objects. The physics objects used in this calculation are produced by a jet-finding algorithm [57, 58] applied to all charged-particle tracks associated to the vertex, plus the corresponding $p_{\mathrm {T}} ^\text {miss}$ computed from those jets. To mitigate the effect of secondary $\mathrm {p}\mathrm {p}$ interactions (pileup), charged-particle tracks associated with vertices other than the primary vertex are not considered for jet clustering or calculating object isolation sums.

Jets are reconstructed by clustering PF candidates using the anti-$k_{\mathrm {T}}$ jet algorithm [57, 58] with a size parameter of 0.4. To eliminate spurious jets, for example those induced by electronics noise, further jet quality criteria [59] are applied. The jet energy response is corrected for the nonlinear response of the detector [60]. There is also a correction to account for the expected contributions of neutral particles from pileup, which cannot be removed based on association with secondary vertices [61]. Jets are required to have $p_{\mathrm {T}} >30\,\text {Ge}\text {V}$ and are restricted to be within $|\eta |< 2.4$. The combined secondary vertex algorithm (CSVv2) at the medium working point [62] is applied to each jet to determine if it should be identified as a bottom quark jet. The CSVv2 algorithm at the specified working point has a 55% efficiency to correctly identify $\mathrm {b}$ jets with $p_{\mathrm {T}} \approx 30\,\text {Ge}\text {V}$. The corresponding misidentification probabilities are 1.6% for gluon and light-flavor quark jets, and 12% for charm quark jets.

Photons with $p_{\mathrm {T}} >100\,\text {Ge}\text {V}$ and $|\eta |<2.4$ are used in this analysis, excluding the ECAL transition region with ${1.44< |\eta |< 1.56}$. To suppress jets erroneously identified as photons from neutral hadron decays, photon candidates are required to be isolated. An isolation cone of radius $\varDelta R = \sqrt{\smash [b]{(\varDelta \phi )^2 + (\varDelta \eta )^2}} < 0.2$ is used, with no dependence on the $p_{\mathrm {T}}$ of the photon candidate. Here, $\phi$ is the azimuthal angle in radians. The energy measured in the isolation cone is corrected for contributions from pileup [61]. The shower shape and the fractions of hadronic and electromagnetic energy associated with the photon candidate are required to be consistent with expectations from prompt photons. The candidates matched to a track measured by the pixel detector (pixel seed) are rejected because they are likely to result from electrons that produced electromagnetic showers.

Similarly, to suppress jets erroneously identified as leptons and genuine leptons from hadron decays, electron and muon candidates are also subjected to isolation requirements. The isolation variable *I* is computed from the scalar $$p_{\mathrm {T}}$$ sum of selected charged hadron, neutral hadron, and photon PF candidates, divided by the lepton $$p_{\mathrm {T}}$$. PF candidates enter the isolation sum if they satisfy $$R < R_{I} (p_{\mathrm {T}})$$. The cone radius $$R_{I} $$ decreases with lepton $$p_{\mathrm {T}}$$ because the collimation of the decay products of the parent particle of the lepton increases with the Lorentz boost of the parent [63]. The values used are $$R_{I} = 0.2$$ for $$p_{\mathrm {T}} ^{\ell } <50\,\text {Ge}\text {V} $$, $$R_{I} = 10\,\text {Ge}\text {V}/p_{\mathrm {T}} ^{\ell } $$ for $$50\le p_{\mathrm {T}} ^{\ell } \le 200\,\text {Ge}\text {V} $$, and $$R_{I} = 0.05$$ for $$p_{\mathrm {T}} ^{\ell } >200\,\text {Ge}\text {V} $$, where $$\ell =\mathrm {e},\mathrm {\mu }$$. As with photons, the expected contributions from pileup are subtracted from the isolation variable. The isolation requirement is $${I<0.1\,(0.2)}$$ for electrons (muons).

We additionally veto events if they contain PF candidates which are identified as an electron, a muon, or a charged hadron, and satisfy an isolation requirement computed using tracks. Isolated hadronic tracks are common in background events with a tau lepton that decays hadronically. The track isolation variable $I_{\text {track}}$ is computed for each candidate from the scalar $p_{\mathrm {T}}$ sum of selected other charged-particle tracks, divided by the candidate $p_{\mathrm {T}}$. Other charged-particle tracks are selected if they lie within a cone of radius 0.3 around the candidate direction and come from the primary vertex. The isolation variable must satisfy $I_{\text {track}} < 0.2$ for electrons and muons, and $I_{\text {track}} < 0.1$ for charged hadrons. Isolated tracks are required to satisfy $|\eta |<2.4$, and the transverse mass of each isolated track with $p_{\mathrm {T}} ^\text {miss}$, $m_{\mathrm {T}} =\sqrt{\smash [b]{2p_{\mathrm {T}} ^{\text {track}} p_{\mathrm {T}} ^\text {miss} (1-\cos {\varDelta \phi })}}$ where $\varDelta \phi$ is the difference in $\phi$ between ${\vec p}_{\mathrm {T}} ^{\text {track}}$ and ${\vec p}_{\mathrm {T}}^{\text {miss}}$, is required to be less than 100$\,\text {Ge}\text {V}$.

Signal event candidates were recorded by requiring a photon at the trigger level with a requirement ${p_{\mathrm {T}} ^{\gamma } >90\,\text {Ge}\text {V}}$ if ${H_{\mathrm {T}} ^{\gamma } =p_{\mathrm {T}} ^{\gamma } +\varSigma p_{\mathrm {T}} ^{\text {jet}} >600\,\text {Ge}\text {V}}$ and ${p_{\mathrm {T}} ^{\gamma } >165\,\text {Ge}\text {V}}$ otherwise. These quantities are computed at the trigger level. The efficiency of this trigger, as measured in data, is $(98\pm 2)\%$ after applying the selection criteria described below. Additional triggers, requiring the presence of charged leptons, photons, or minimum $H_{\mathrm {T}} =\varSigma p_{\mathrm {T}} ^{\text {jet}}$, are used to select control samples employed in the evaluation of backgrounds.

Signal-like candidate events must fulfill one of two requirements, based on the trigger criteria described above: ${p_{\mathrm {T}} ^{\gamma } >100\,\text {Ge}\text {V}}$ and ${H_{\mathrm {T}} ^{\gamma } >800\,\text {Ge}\text {V}}$, or $p_{\mathrm {T}} ^{\gamma } >190\,\text {Ge}\text {V}$ and $H_{\mathrm {T}} ^{\gamma } >500\,\text {Ge}\text {V}$. In addition to these requirements, the events should have at least 2 jets and $p_{\mathrm {T}} ^\text {miss} >100\,\text {Ge}\text {V}$. To reduce backgrounds from the SM processes that produce a leptonically decaying $\mathrm {W}$ boson, resulting in $p_{\mathrm {T}} ^\text {miss}$ from the undetected neutrino, events are rejected if they have any charged light leptons ($\mathrm {e}$, $\mathrm {\mu }$) with $p_{\mathrm {T}} >10\,\text {Ge}\text {V}$ or any isolated electron, muon, or charged hadron tracks with ${p_{\mathrm {T}} >5,5,10\,\text {Ge}\text {V}}$, respectively. Events from the ${\gamma }\text {+jets}$ process typically satisfy the above criteria when the energy of a jet is mismeasured, inducing artificial $p_{\mathrm {T}} ^\text {miss}$. To reject these events, the two highest $p_{\mathrm {T}}$ jets are both required to have an angular separation from the $p_{\mathrm {T}} ^\text {miss}$ direction in the transverse plane, $\varDelta \phi _{1,2}>0.3$. Events with reconstruction failures, detector noise, or beam halo interactions are rejected using dedicated identification requirements [64].

The selected events are divided into 25 exclusive signal regions, also called signal bins, based on $p_{\mathrm {T}} ^\text {miss}$, the number of jets $N_{\text {jets}}$, and the number of $\mathrm {b}$-tagged jets $N_{{\mathrm {b}}\text {-jets}}$. The signal regions can be grouped into 6 categories based on $N_{\text {jets}}$ and $N_{{\mathrm {b}}\text {-jets}}$, whose intervals are defined to be $N_{\text {jets}}$: 2–4, 5–6, ${\ge }7$; and $N_{{\mathrm {b}}\text {-jets}}$: 0, ${\ge }1$. Within each of the 6 categories, events are further distinguished based on 4 exclusive regions, defined as: ${200<p_{\mathrm {T}} ^\text {miss} <270}$, ${270<p_{\mathrm {T}} ^\text {miss} <350}$, ${350<p_{\mathrm {T}} ^\text {miss} <450}$, and ${p_{\mathrm {T}} ^\text {miss} >450\,\text {Ge}\text {V}}$. In the lowest $N_{\text {jets}}$, $N_{{\mathrm {b}}\text {-jets}}$ category, the highest $p_{\mathrm {T}} ^\text {miss}$ bin is further subdivided into two intervals: ${450<p_{\mathrm {T}} ^\text {miss} <750}$ and ${p_{\mathrm {T}} ^\text {miss} >750\,\text {Ge}\text {V}}$. Events with ${100<p_{\mathrm {T}} ^\text {miss} <200\,\text {Ge}\text {V}}$ are used as a control region for estimating SM backgrounds. These categories in $N_{\text {jets}}$, $N_{{\mathrm {b}}\text {-jets}}$, and $p_{\mathrm {T}} ^\text {miss}$ were found to provide good sensitivity to the various signal models described above, while minimizing uncertainties in the background predictions.

## Background estimation

There are four main mechanisms by which SM processes can produce events with the target signature of a photon, multiple jets, and $p_{\mathrm {T}} ^\text {miss}$. These mechanisms are: (1) the production of a high-$p_{\mathrm {T}}$ photon along with a $\mathrm {W}$ or $\mathrm {Z}$ boson that decays leptonically, and either any resulting electron or muon is “lost” (lost-lepton) or any resulting $\mathrm {\tau }$ lepton decays hadronically ($\tau _\mathrm {h}$); (2) the production of a $\mathrm {W}$ boson that decays to $\mathrm {e}\nu$ and the electron is misidentified as a photon; (3) the production of a high-$p_{\mathrm {T}}$ photon in association with a $\mathrm {Z}$ boson that decays to neutrinos; and (4) the production of a photon along with a jet that is mismeasured, inducing high $p_{\mathrm {T}} ^\text {miss}$. QCD multijet events with a jet misidentified as a photon and a mismeasured jet do not contribute significantly to the SM background.

The total event yield from each source of background is estimated separately for each of the 25 signal regions. The methods and uncertainties associated with the background predictions are detailed in the following sections.

### Lost-lepton and $\tau _\mathrm {h}$ backgrounds

The lost-lepton background arises from events in which the charged lepton from a leptonically decaying $\mathrm {W}$ boson, produced directly or from the decay of a top quark, cannot be identified. This can occur because the lepton is out of acceptance, fails the identification requirements, or fails the isolation requirements. For example, in events with high-$p_{\mathrm {T}}$ top quarks, the top quark decay products will be collimated, forcing the $\mathrm {b}$ jet to be closer to the charged lepton. In this case, the lepton is more likely to fail the isolation requirements. This background is estimated by studying control regions in both data and simulation, obtained by requiring both a well-identified photon and a light lepton ($\mathrm {e}$, $\mathrm {\mu }$). For every signal region, there are two lost lepton control regions that have the exact same definition as the signal region except either exactly one electron or exactly one muon is required.

The $\tau _\mathrm {h}$ background arises from events in which a $\mathrm {W}$ boson decays to a $\mathrm {\tau }$ lepton, which subsequently decays to mesons and a neutrino. These hadronic decays of $\mathrm {\tau }$ leptons occur approximately $65\%$ of the time. Because of lepton universality, the fraction of events with $\tau _\mathrm {h}$ candidates can be estimated from the yield of events containing a single muon, after correcting for the reconstruction differences and for the $\tau _\mathrm {h}$ branching fraction.

The lost-lepton and $\tau _\mathrm {h}$ background predictions rely on an extrapolation between $\mathrm {e}{\gamma }$ or $\mathrm {\mu }{\gamma }$ event yields and single photon event yields. In all control regions where a single light lepton is required, the dominant SM processes that contribute are $\mathrm {W}{\gamma }$ and ${\mathrm {t}\overline{\mathrm {t}}} {\gamma }$. Lost-muon and hadronic tau events are estimated using $\mathrm {\mu }{\gamma }$ control regions, while lost-electron events are estimated using $\mathrm {e}{\gamma }$ control regions. In each control region, exactly one electron or muon is required and the isolated track veto for the selected lepton flavor is removed. In order to reduce the effect of signal contamination and to increase the fraction of SM events in the control sample, events are only selected if the $m_{\mathrm {T}}$ of the lepton-$p_{\mathrm {T}} ^\text {miss}$ system is less than 100$\,\text {Ge}\text {V}$. In SM background events with a single lepton and $p_{\mathrm {T}} ^\text {miss}$, the $m_{\mathrm {T}}$ of the system is constrained by the mass of the $\mathrm {W}$ boson; this is not the case for signal events, because of the presence of gravitinos. All other kinematic variable requirements for each signal region are applied to the corresponding control regions.

**Fig. 2:**
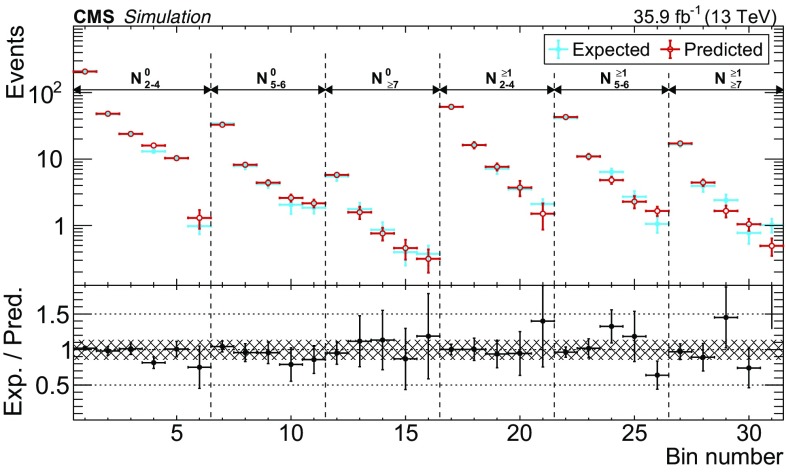
The lost-lepton and $\tau _\mathrm {h}$ event yields as predicted directly from simulation in the signal regions, shown in red, and from the prediction procedure applied to simulated $\mathrm {e}{\gamma }$ or $\mathrm {\mu }{\gamma }$ events, shown in blue. The error bars correspond to the statistical uncertainties from the limited number of events in simulation. The bottom panel shows the ratio of the simulation expectation (Exp.) and the simulation-based prediction (Pred.). The hashed area shows the expected uncertainties from data-to-simulation correction factors, PDFs, and renormalization and factorization scales. The categories, denoted by dashed lines, are labeled as $N_{\mathrm {j}}^{\mathrm {b}}$, where j refers to the number of jets and b refers to the number of $\mathrm {b}$-tagged jets. The numbered bins within each category are the various $p_{\mathrm {T}} ^\text {miss}$ bins. In each of these regions, the first bin corresponds to $100<p_{\mathrm {T}} ^\text {miss} <200\,\text {Ge}\text {V}$, which belongs to a control region. The remaining bins correspond to the signal regions in Table 1

Transfer factors are derived using simulated $\mathrm {W}$ ${\gamma }\text {+jets}$ and ${\mathrm {t}\overline{\mathrm {t}}} {\gamma }$ processes, which determine the average number of events expected in the signal region for each $\mathrm {e}{\gamma }$ or $\mathrm {\mu }{\gamma }$ event observed in the control region. The $\mathrm {Z}{\gamma }$ events in which the $\mathrm {Z}$ boson decays leptonically have a negligible contribution to the transfer factors. The transfer factors applied to the $\mathrm {\mu }{\gamma }$ control regions account for both lost-$\mathrm {\mu }$ events and $\tau _\mathrm {h}$ events. They are denoted by the symbol $T_{\mathrm {\mu },\mathrm {\tau }}$ and are typically in the range ${0.7<T_{\mathrm {\mu },\mathrm {\tau }} <1.0}$. The transfer factors applied to $\mathrm {e}{\gamma }$ events account for only the lost-$\mathrm {e}$ events. They are denoted by the symbol $T_{\mathrm {e}}$ and are typically in the range ${0.3<T_{\mathrm {e}} <0.6}$. The transfer factors are parameterized versus $N_{\text {jets}}$, $N_{{\mathrm {b}}\text {-jets}}$, and $p_{\mathrm {T}} ^\text {miss}$; however, for ${p_{\mathrm {T}} ^\text {miss} >150\,\text {Ge}\text {V}}$, $T_{\ell }$ is found to be independent of $p_{\mathrm {T}} ^\text {miss}$. The parameterization of the transfer factors is validated using simulation by treating $\mathrm {e}{\gamma }$ or $\mathrm {\mu }{\gamma }$ events like data and comparing the predicted lost-lepton and $\tau _\mathrm {h}$ event yields to the true simulated event yields in the signal regions. This comparison is shown in Fig. 2. The prediction in each signal region is ${N_{\ell }^{\text {pred}} = \varSigma _{i} N_{i} T_{\ell ,i}}$, where ${\ell =\mathrm {e},\mathrm {\mu }}$ and *i* ranges from 1 to *n*, where *n* is the number of transfer factors that contribute in a given signal region.

The dominant uncertainty in the lost-lepton background predictions arises from the limited numbers of events in the $\mathrm {e}{\gamma }$ and $\mathrm {\mu }{\gamma }$ control regions. These uncertainties are modeled in the final statistical interpretations as a gamma distribution whose shape parameter is set by the observed number of events and whose scale parameter is the average transfer factor for that bin. Other systematic uncertainties in the determination of the transfer factors include the statistical uncertainty from the limited number of simulated events, which is typically 5–10% but can be as large as 20%, as well as uncertainties in the jet energy corrections, PDFs, renormalization ($\mu _{\text {R}}$) and factorization ($\mu _{\text {F}}$) scales, and simulation correction factors. The uncertainties in $\mu _{\text {R}}$ and $\mu _{\text {F}}$ are obtained by varying each value independently by factors of 0.5 and 2.0 [65, 66]. Simulation correction factors are used to account for differences between the observed data and modeling of $\mathrm {b}$-tagging efficiencies, $\mathrm {b}$ jet misidentification, and lepton reconstruction efficiencies in simulation. One of the largest uncertainties, apart from the statistical uncertainty in the data control regions and the simulation, comes from mismodeling of photons which are collinear with electrons, which has a 12% effect on the lost-lepton prediction.

### Misidentified photon background

Events containing the decay $\mathrm {W}\rightarrow \mathrm {e}\nu$ are the primary source of electrons that are erroneously identified as photons. Photon misidentification can occur when a pixel detector seed fails to be associated with the energy deposit in the ECAL. Given a misidentification rate, which relates events with an erroneously identified photon to events with a well-identified electron, the photon background can be estimated from a single-electron (zero-photon) control region. The misidentification rate is estimated in simulation and corrections are derived from observed data to account for any mismodeling in simulation.

The single-electron control regions are defined by the same kinematic requirements as the single-photon signal regions, except that we require no photons and exactly one electron, and we use the momentum of the electron in place of the momentum of the photon for photon-based variables. As explained in the previous section, in addition to all of the signal region selections, events are required to satisfy ${m_{\mathrm {T}} (\mathrm {e},p_{\mathrm {T}} ^\text {miss})<100\,\text {Ge}\text {V}}$.

To extrapolate from the event yields in the single-electron control regions to the event yields for the misidentified photon background in the signal regions, we derive a misidentification rate $f=N_{\gamma }/N_{\mathrm {e}}$ using a combination of simulation and data. The misidentification rate is determined as a function of the electron $p_{\mathrm {T}}$ and the multiplicity $Q_{\text {mult}}$ of charged-particle tracks from the primary vertex in a region around the electron candidate. The charged-track multiplicity is computed by counting the number of charged PF candidates (electrons, muons, hadrons) in the jet closest to the electron candidate. If there is no jet within $\varDelta R < 0.3$ of the electron candidate, $Q_{\text {mult}}$ is set to zero. A typical event in the single-electron control region has a $Q_{\text {mult}}$ of 3–4. The electron $p_{\mathrm {T}}$ and $Q_{\text {mult}}$ dependence of the misidentification rate is derived using simulated $\mathrm {W}$+jets and ${\mathrm {t}\overline{\mathrm {t}}}$+jets events. The misidentification rate is on average 1–2%, but can be as low as 0.5% for events with high $Q_{\text {mult}}$.

To account for systematic differences between the misidentification rates in data and simulation, we correct the misidentification rate by measuring it in both simulated and observed Drell–Yan (DY) events. Separate corrections are derived for low $Q_{\text {mult}}$ (${\le }1$) and high $Q_{\text {mult}}$ (${\ge }2$). The DY control region is defined by requiring one electron with ${p_{\mathrm {T}} >40\,\text {Ge}\text {V}}$ and another reconstructed particle, either a photon or an oppositely charged electron, with ${p_{\mathrm {T}} >100\,\text {Ge}\text {V}}$. A further requirement ${50<(m_{\mathrm {e}^+\mathrm {e}^-}\,\text {or}\,m_{\mathrm {e}{\gamma }})<130\,\text {Ge}\text {V}}$ is applied to ensure the particles are consistent with the decay products of a $\mathrm {Z}$ boson, and therefore the photon is likely to be a misidentified electron. The misidentification rate is computed as the ratio $N_{\mathrm {e}{\gamma }}/N_{\mathrm {e}^+\mathrm {e}^-}$, where $N_{\mathrm {e}{\gamma }}$ ($N_{\mathrm {e}^+\mathrm {e}^-}$) is the number of events in the $\mathrm {e}{\gamma }$ ($\mathrm {e}^+\mathrm {e}^-$) control region. It is found to be 15–20% higher in data than in simulation.

The prediction of the misidentified-photon background in the signal region is then given by the weighted sum of the observed events in the control region, where the weight is given by the data-corrected misidentification rate for photons. The dominant uncertainty in the prediction is a 14% uncertainty in the data-to-simulation correction factors, followed by the uncertainty in the limited number of events in the simulation at large values of $p_{\mathrm {T}} ^\text {miss}$. The misidentified-photon background prediction also includes uncertainties in the modeling of initial-state radiation (ISR) in the simulation, statistical uncertainties from the limited number of events in the data control regions, uncertainties in the pileup modeling, and uncertainties in the trigger efficiency measurement.

### Background from $\mathrm {Z}(\nu \overline{\nu })\gamma$ events

Decays of the $\mathrm {Z}$ boson to invisible particles constitute a major background for events with low $N_{\text {jets}}$, low $N_{{\mathrm {b}}\text {-jets}}$, and high $p_{\mathrm {T}} ^\text {miss}$. The $\mathrm {Z}(\nu \overline{\nu })\gamma$ background is estimated using $\mathrm {Z}(\ell ^{+}\ell ^{-})\gamma$ events. The shape of the distribution of $p_{\mathrm {T}} ^\text {miss}$ vs. $N_{\text {jets}}$ in $\mathrm {Z}(\nu \overline{\nu })\gamma$ events is modeled in simulation, while the normalization and the purity of the control region are measured in data.

Events in the $\ell ^{+}\ell ^{-}\gamma$ control region are required to have exactly two oppositely charged, same-flavor leptons ($\ell = \mathrm {e}$ or $\mathrm {\mu }$) and one photon with $p_{\mathrm {T}} >100\,\text {Ge}\text {V}$. The dilepton invariant mass $m_{\ell \ell }$ is required to be consistent with the $\mathrm {Z}$ boson mass, $80<m_{\ell \ell } <100\,\text {Ge}\text {V}$. The charged leptons serve as a proxy for neutrinos, so the event-level kinematic variables, such as $p_{\mathrm {T}} ^\text {miss}$, are calculated after removing charged leptons from the event.

The $\ell ^{+}\ell ^{-}\gamma$ control region may contain a small fraction of events from processes other than $\mathrm {Z}(\ell ^{+}\ell ^{-})\gamma$, primarily ${\mathrm {t}\overline{\mathrm {t}}}$ $\gamma$. We define the purity of the control region as the percentage of events originating from the $\mathrm {Z}(\ell ^{+}\ell ^{-})\gamma$ process. The purity is computed in data by measuring the number of events in the corresponding oppositely charged, different-flavor control region, which has a higher proportion of ${\mathrm {t}\overline{\mathrm {t}}} {\gamma }$ events. The purity is found to be $(97\pm 3)\%$. A statistically compatible purity is also measured in the oppositely charged, same-flavor control region. In this region, the $m_{\ell \ell }$ distribution is used to extrapolate from the number of events with $m_{\ell \ell }$ far from the $\mathrm {Z}$ boson mass to the number of events with $m_{\ell \ell }$ close to it.

The $\mathrm {Z}(\nu \overline{\nu })\gamma$ predictions from simulation are scaled to the total $\mathrm {Z}(\ell ^{+}\ell ^{-})\gamma$ yield observed according to ${N_{\mathrm {Z}(\nu \overline{\nu })\gamma } = \beta R_{\nu \nu /\ell \ell } N_{\mathrm {Z}(\ell ^{+}\ell ^{-})\gamma }}$, where $\beta$ is the purity of the $\mathrm {Z}(\ell ^{+}\ell ^{-})\gamma$ control region and $R_{\nu \nu /\ell \ell }$ is the ratio between the expected number of $\mathrm {Z}(\nu \overline{\nu })\gamma$ and $\mathrm {Z}(\ell ^{+}\ell ^{-})\gamma$ events. The ratio $R_{\nu \nu /\ell \ell }$, which accounts for lepton reconstruction effects and the relative branching fractions for $\mathrm {Z}\rightarrow \nu \overline{\nu }$ and $\mathrm {Z}\rightarrow \ell ^{+}\ell ^{-}$, is determined from simulation.

The primary uncertainty in the $\mathrm {Z}(\nu \overline{\nu })\gamma$ prediction arises from uncertainties in the $p_{\mathrm {T}} ^\text {miss}$ distribution from the simulation. Other uncertainties include statistical uncertainties from the limited number of events in the simulation and uncertainties in the estimation of the control region purity. The $p_{\mathrm {T}} ^{\gamma }$-dependent NLO electroweak corrections [67] are assigned as additional uncertainties to account for any mismodeling of the photon $p_{\mathrm {T}}$ in simulation. This uncertainty has a magnitude of 8% for the lowest $p_{\mathrm {T}} ^\text {miss}$ bin and rises to 40% for $p_{\mathrm {T}} ^\text {miss} >750\,\text {Ge}\text {V}$.

### Background from $\gamma$+jets events

The ${\gamma }\text {+jets}$ background is dominated by events in which a genuine photon is accompanied by an energetic jet with mismeasured $p_{\mathrm {T}}$, resulting in high $p_{\mathrm {T}} ^\text {miss}$. The QCD multijet events with a jet misidentified as a photon and a mismeasured jet contribute to this background at a much smaller rate; these events are measured together with events from the ${\gamma }\text {+jets}$ process. Most of these events are removed by requiring that the azimuthal angles between the ${\vec p}_{\mathrm {T}}^{\text {miss}}$ and each of the two highest $p_{\mathrm {T}}$ jets satisfy $\varDelta \phi _{1,2}>0.3$. Inverting this requirement provides a large control region of low-$\varDelta \phi$ events that is used to predict the ${\gamma }\text {+jets}$ background in the signal regions. The ratio of high-$\varDelta \phi$ events to low-$\varDelta \phi$ events, $R_{\text {h/l}}$, is derived from the low-$p_{\mathrm {T}} ^\text {miss}$ sideband ($100<p_{\mathrm {T}} ^\text {miss} <200\,\text {Ge}\text {V}$).

While most of the events in both the low-$\varDelta \phi$ and the low-$p_{\mathrm {T}} ^\text {miss}$ control regions are ${\gamma }\text {+jets}$ events, electroweak backgrounds in which $p_{\mathrm {T}} ^\text {miss}$ arises from $\mathrm {W}$ or $\mathrm {Z}$ bosons decaying to one or more neutrinos, like those discussed previously, will contaminate these control regions. The contamination can be significant for high $N_{\text {jets}}$ and $N_{{\mathrm {b}}\text {-jets}}$, where ${\mathrm {t}\overline{\mathrm {t}}}$ events are more prevalent. The rates of these events in the control regions are predicted using the same techniques, as discussed in the previous sections.

**Fig. 3:**
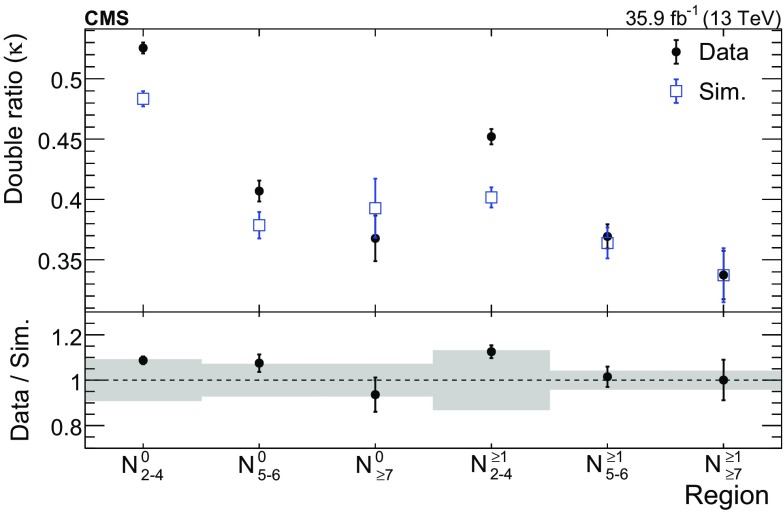
The double ratio $\kappa$ in each $N_{\text {jets}}$-$N_{{\mathrm {b}}\text {-jets}}$ region for zero-photon events. The filled black circles are the observed $\kappa$ values after subtracting the electroweak contamination based on simulation. The open blue squares are the $\kappa$ values computed directly from simulation. The ratio is shown in the bottom panel, where the shaded region corresponds to the systematic uncertainty in the ${\gamma }\text {+jets}$ prediction. In the label $N_{\mathrm {j}}^{\mathrm {b}}$, j refers to the number of jets and b refers to the number of $\mathrm {b}$-tagged jets

A double ratio $\kappa =R_{\text {h/l}} ^{p_{\mathrm {T}} ^\text {miss} >200\,\text {Ge}\text {V}}/R_{\text {h/l}} ^{p_{\mathrm {T}} ^\text {miss} <200\,\text {Ge}\text {V}}$ is derived from simulated ${\gamma }\text {+jets}$ events in order to account for the dependence of $R_{\text {h/l}}$ on $p_{\mathrm {T}} ^\text {miss}$. To test how well the simulation models $\kappa$, we use a zero-photon validation region in which the contribution from events containing a mismeasured jet dominates. To be consistent with the trigger used to select the data in this region, these events are also required to have $H_{\mathrm {T}} >1000\,\text {Ge}\text {V}$. Electroweak contamination in the zero-photon validation region is estimated using simulated $\mathrm {V}$ ${\gamma }\text {+jets}$ ($\mathrm {V}$ = $\mathrm {Z}$, $\mathrm {W}$), ${\mathrm {t}\overline{\mathrm {t}}} {\gamma }$, ${\mathrm {t}\overline{\mathrm {t}}}$+jets, $\mathrm {W}$+jets, and $\mathrm {Z}(\nu \overline{\nu })$+jets events. The comparison of $\kappa$ in data and simulation is shown in Fig. 3. The level of disagreement is found to be less than 20%.

Event yields for the ${\gamma }\text {+jets}$ background are computed from the high-$p_{\mathrm {T}} ^\text {miss}$, low-$\varDelta \phi$ control regions according to $N_{{\gamma }\text {+jets}} = \kappa N_{\text {low-}\varDelta \phi }R_{\text {h/l}}$. $N_{\text {low-}\varDelta \phi }$ is the event yield in the high-$p_{\mathrm {T}} ^\text {miss}$, low-$\varDelta \phi$ control region after removing contributions from electroweak backgrounds.

Uncertainties in the ${\gamma }\text {+jets}$ prediction are dominated by the statistical uncertainties either from the limited number of events in the low-$\varDelta \phi$ control regions or from the predictions of the electroweak contamination. The ${<}20\%$ disagreement between the $\kappa$ values in data and simulation in the zero-photon validation region is included as an additional uncertainty. Uncertainties in the $\mathrm {b}$-tagging correction factors are a minor contribution to the uncertainty in the ${\gamma }\text {+jets}$ prediction.

## Results and interpretations

**Fig. 4:**
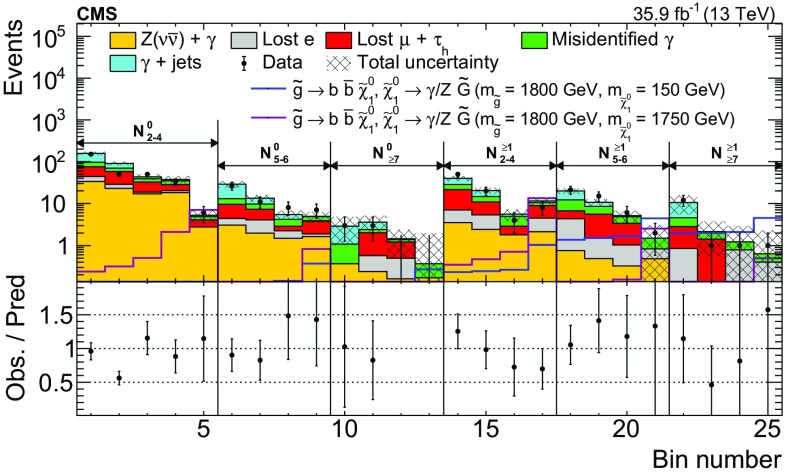
Observed numbers of events and predicted numbers of events from the various SM backgrounds in the 25 signal regions. The categories, denoted by vertical lines, are labeled as $N_{\mathrm {j}}^{\mathrm {b}}$, where j refers to the number of jets and b refers to the number of $\mathrm {b}$-tagged jets. The numbered bins within each category are the various $p_{\mathrm {T}} ^\text {miss}$ bins, as defined in Table 1. The lower panel shows the ratio of the observed events to the predicted SM background events. The error bars in the lower panel are the quadrature sum of the statistical uncertainty in the observed data and the systematic uncertainty in the predicted backgrounds before the adjustments based on a maximum likelihood fit to data assuming no signal strength

The predicted background and observed yields are shown in Table 1 and Fig. 4. The largest deviation is found in bin 2 ($2\le N_{\text {jets}} \le 4$, $N_{{\mathrm {b}}\text {-jets}} =0$, and $270<p_{\mathrm {T}} ^\text {miss} <350\,\text {Ge}\text {V}$), where the background is predicted to be 91 events with 51 events observed. The local significance of this single bin was computed to be around 2 standard deviations below the SM expectation. This calculation does not account for the look-elsewhere effect associated with the use of 25 exclusive signal regions, which is expected to reduce this significance. In general, a large deviation in a single bin is inconsistent with the expected distributions of events from the signal models considered here. The observations in all other bins are consistent with the SM expectations within one standard deviation.

**Table 1:** Predicted and observed event yields for each of the 25 exclusive signal regions

$N_{\text {jets}}$	$N_{{\mathrm {b}}\text {-jets}}$	$p_{\mathrm {T}} ^\text {miss}$ (GeV)	Lost $\mathrm {e}$	Lost $\mathrm {\mu }$ + $\tau _\mathrm {h}$	Misid. $\gamma$	$\mathrm {Z}(\nu \overline{\nu })\gamma$	${\gamma }\text {+jets}$	Total	Data
2–4	0	200–270	10.5 ± 2.6	31.2 ± 6.0	22.3 ± 5.4	33.6 ± 8.3	60 ± 11	157 ± 16	151
2–4	0	270–350	5.8 ± 1.8	29.6 ± 5.9	11.9 ± 2.9	22.9 ± 6.0	20.5 ± 4.3	91 ± 10	51
2–4	0	350–450	1.68 ± 0.88	13.9 ± 3.9	6.6 ± 1.6	17.0 ± 5.2	4.1 ± 1.4	43.3± 6.8	50
2–4	0	450–750	1.98 ± 0.94	8.1 ± 3.1	6.7 ± 1.5	18.1 ± 7.1	2.5 ± 1.3	37.4± 8.0	33
2–4	0	${>} \, 750$	$0.00_{-0.00}^{+0.69}$	1.2 ± 1.2	0.79 ± 0.19	2.8 ± 1.2	$0.41_{-0.41}^{+0.42}$	5.2 ± 1.9	6
5–6	0	200–270	1.28 ± 0.61	5.1 ± 1.9	3.53 ± 0.75	3.09 ± 0.78	15.8 ± 4.8	28.8 ± 5.3	26
5–6	0	270–350	2.06 ± 0.80	3.2 ± 1.5	2.39 ± 0.56	1.98 ± 0.54	3.7 ± 1.8	13.3 ± 2.6	11
5–6	0	350–450	0.77 ± 0.46	$0.64_{-0.64}^{+0.65}$	1.26 ± 0.30	1.49 ± 0.47	1.23 ± 0.97	5.4 ± 1.4	8
5–6	0	${>} \, 450$	0.26 ± 0.26	1.9 ± 1.1	1.00 ± 0.24	1.65 ± 0.65	$0.07_{-0.07}^{+0.52}$	4.9 ± 1.4	7
${\ge } \, 7$	0	200–270	$0.00_{-0.00}^{+0.61}$	$0.0_{-0.0}^{+1.3}$	0.72 ± 0.16	0.37 ± 0.11	1.8 ± 1.2	2.9 ± 1.9	3
${\ge } \, 7$	0	270–350	$0.34_{-0.34}^{+0.35}$	1.5 ± 1.0	0.38 ± 0.10	0.24 ± 0.08	1.22 ± 0.94	3.6 ± 1.5	3
${\ge } \, 7$	0	350–450	$0.34_{-0.34}^{+0.35}$	0.73 ± 0.73	0.17 ± 0.05	0.16 ± 0.07	$0.07_{-0.07}^{+0.50}$	1.46 ± 0.96	0
${\ge } \, 7$	0	${>} \, 450$	$0.00_{-0.00}^{+0.61}$	$0.0_{-0.0}^{+1.3}$	0.20 ± 0.06	0.17 ± 0.08	$0.00_{-0.00}^{+0.75}$	$0.37_{-0.37}^{+1.60}$	0
2–4	${\ge }1$	200–270	3.4 ± 1.5	14.5 ± 4.2	7.1 ± 1.7	3.55 ± 0.89	11.3 ± 3.3	39.8 ± 5.9	50
2–4	${\ge }1$	270–350	2.9 ± 1.4	5.6 ± 2.5	3.79 ± 0.92	2.45 ± 0.65	5.7 ± 1.8	20.4 ± 3.6	20
2–4	${\ge }1$	350–450	$0.0_{-0.0}^{+1.0}$	1.1 ± 1.1	2.00 ± 0.45	1.81 ± 0.55	0.59 ± 0.44	5.5 ± 1.7	4
2–4	${\ge }1$	${>} \, 450$	2.3 ± 1.2	4.4 ± 2.3	1.62 ± 0.38	2.14 ± 0.84	0.95 ± 0.54	11.5 ± 2.8	8
5–6	${\ge }1$	200–270	3.5 ± 1.3	2.4 ± 1.4	5.5 ± 1.2	0.76 ± 0.20	7.7 ± 2.4	19.9 ± 3.3	21
5–6	${\ge }1$	270–350	1.06 ± 0.64	4.0 ± 1.8	2.98 ± 0.63	0.49 ± 0.14	2.1 ± 1.0	10.6 ± 2.3	15
5–6	${\ge }1$	350–450	0.71 ± 0.51	2.4 ± 1.4	1.38 ± 0.29	0.32 ± 0.11	$0.30_{-0.30}^{+0.49}$	5.1 ± 1.6	6
5–6	${\ge }1$	${>} \, 450$	$0.35_{-0.35}^{+0.36}$	$0.0_{-0.0}^{+1.4}$	0.67 ± 0.15	0.48 ± 0.20	$0.00_{-0.00}^{+0.56}$	$1.5_{-1.5}^{+1.6}$	2
${\ge } \, 7$	${\ge }1$	200–270	0.72 ± 0.53	2.0 ± 1.2	1.68 ± 0.37	0.13 ± 0.04	5.9 ± 5.0	10.5 ± 5.1	12
${\ge } \, 7$	${\ge }1$	270–350	$0.00_{-0.00}^{+0.65}$	1.33 ± 0.96	0.73 ± 0.16	0.10 ± 0.04	$0.0_{-0.0}^{+1.1}$	2.2 ± 1.6	1
${\ge } \, 7$	${\ge }1$	350–450	0.72 ± 0.53	$0.0_{-0.0}^{+1.2}$	0.44 ± 0.10	0.07 ± 0.03	$0.0_{-0.0}^{+1.1}$	$1.2_{-1.2}^{+1.7}$	1
${\ge } \, 7$	${\ge }1$	${>} \, 450$	$0.36_{-0.36}^{+0.37}$	$0.0_{-0.0}^{+1.2}$	0.23 ± 0.07	0.04 ± 0.02	$0.0_{-0.0}^{+1.1}$	$0.6_{-0.6}^{+1.7}$	1

Limits are evaluated for the production cross sections of the signal scenarios discussed in Sect. 1 using a maximum likelihood fit for the SUSY signal strength, the yields of the five classes of background events shown in Fig. 4, and various nuisance parameters. The SUSY signal strength $\mu$ is defined to be the ratio of the observed signal cross section to the predicted cross section. A nuisance parameter refers to a variable not of interest in this search, such as the effect of parton distribution function uncertainties in a background prediction. The nuisance parameters are constrained by observed data in the fit. The uncertainties in the predicted signal yield arise from the uncertainties in renormalization and factorization scales, ISR modeling, jet energy scale, $\mathrm {b}$-tagging efficiency and misidentification rate, corrections to simulation, limited numbers of simulated events, and the integrated luminosity measurement [26]. The largest uncertainty comes from the ISR modeling; it ranges from 4 to 30% depending on the signal region and the signal parameters, taking higher values for regions with large $N_{\text {jets}}$ or for signals with $\varDelta m \approx 0$. Here, $\varDelta m$ is the difference in mass between the gluino or squark and its decay products, e.g. $\varDelta m = m_{\widetilde{\mathrm {g}}}- (m_{\widetilde{\chi }^{0}_{1}} + 2m_{\mathrm {t}})$ for the T5ttttZG model when on-shell top quarks are produced. The second-largest uncertainty comes from the correction for differences between Geant4 and the fast simulation in $p_{\mathrm {T}} ^\text {miss}$ modeling, with a maximum value of 10%. The procedures used to evaluate the systematic uncertainties in the signal predictions in the context of this search are described in Ref. [68].

For the models of gluino pair production considered here, the limits are derived as a function of $m_{\widetilde{\mathrm {g}}}$ and $m_{\widetilde{\chi }^{0}_{1}}$, while for the model of top squark pair production, the limits are a function of $m_{\widetilde{\mathrm {t}}}$ and $m_{\widetilde{\chi }^{0}_{1}}$. The likelihood used for the statistical interpretation models the yield in each of the signal regions as a Poisson distribution, multiplied by constraints which account for the uncertainties in the background predictions and signal yields. For the predictions in which an observed event yield in a control region is scaled, a gamma distribution is used to model the Poisson uncertainty of the observed control region yield. All other uncertainties are modeled as log-normal distributions. The test statistic is $q_{\mu }=-2\ln {\mathcal {L}_{\mu }/\mathcal {L}_{\text {max}}}$, where $\mathcal {L}_{\text {max}}$ is the maximum likelihood determined by leaving all parameters as free, including the signal strength, and $\mathcal {L}_{\mu }$ is the maximum likelihood for a fixed value of $\mu$. Limits are determined using an approximation of the asymptotic form of the test statistic distribution [69] in conjunction with the $\text {CL}_\text {s}$ criterion [70, 71]. Expected upper limits are derived by varying observed yields according to the expectations from the background-only hypothesis.

**Fig. 5:**
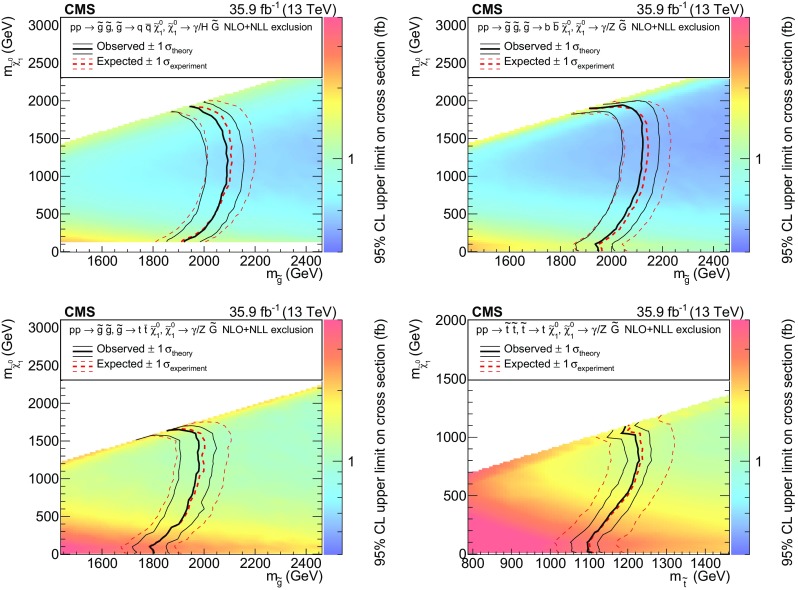
Observed and expected 95% $\text {CL}$ upper limits for gluino or top squark pair production cross sections for the T5qqqqHG (upper left), T5bbbbZG (upper right), T5ttttZG (bottom left), and T6ttZG (bottom right) models. Black lines denote the observed exclusion limit and the uncertainty due to variations of the theoretical prediction of the gluino or top squark pair production cross section. The dashed lines correspond to the region containing 68% of the distribution of the expected exclusion limits under the background-only hypothesis

Using the statistical procedure described above, 95% confidence level ($\text {CL}$) upper limits are computed on the signal cross section for each simplified model and each mass hypothesis. Exclusion limits are defined by comparing observed upper limits to the predicted NLO+NLL signal cross section. The signal cross sections are also varied according to theoretical uncertainties to give a ${\pm }1$ standard deviation variation on the observed exclusion contour. The 95% $\text {CL}$ cross section limits and exclusion contours for the four models considered, T5qqqqHG, T5bbbbZG, T5ttttZG, and T6ttZG, are shown in Fig. 5.

Generally, the limits degrade at both high and low $m_{\widetilde{\chi }^{0}_{1}}$. For $m_{\widetilde{\chi }^{0}_{1}} \approx m_{\widetilde{\mathrm {g}}} \,(m_{\widetilde{\mathrm {t}}})$, the quarks from the decay of gluinos (top squarks) have low $p_{\mathrm {T}}$. Correspondingly, the $H_{\mathrm {T}} ^{\gamma }$, $N_{\text {jets}}$, and $N_{{\mathrm {b}}\text {-jets}}$ distributions tend toward lower values, reducing the signal efficiency and causing signal events to populate regions with higher background yields. For small $m_{\widetilde{\chi }^{0}_{1}}$, the quarks produced in the decay of gluinos or top squarks have high $p_{\mathrm {T}}$ but lower $p_{\mathrm {T}} ^\text {miss}$ on average. For all models except T5qqqqHG, when the NLSP mass drops below the mass of the $\mathrm {Z}$ boson, the kinematics of the NLSP decay require the $\mathrm {Z}$ boson to be far off-shell. As the $\mathrm {Z}$ boson mass is forced to be lower, the LSP will carry a larger fraction of the momentum of the NLSP, producing larger $p_{\mathrm {T}} ^\text {miss}$. This causes a slight increase in the sensitivity when the NLSP mass is near the $\mathrm {Z}$ boson mass. While a similar effect would happen for the T5qqqqHG model, the simulation used here does not probe the region of parameter space where the Higgs boson would be forced to have a mass far off-shell. Similarly, the limits for top squark production improve slightly at very high $m_{\widetilde{\chi }^{0}_{1}}$, when the top quarks become off-shell. In this case, the $\widetilde{\chi }^{0}_{1}$ carries a larger fraction of the top squark momentum, increasing the $p_{\mathrm {T}} ^\text {miss}$.

For moderate $m_{\widetilde{\chi }^{0}_{1}}$, gluino masses as large as 2090, 2120, and 1970$\,\text {Ge}\text {V}$ are excluded for the T5qqqqHG, T5bbbbZG, and T5ttttZG models, respectively. Top squark masses as large as 1230$\,\text {Ge}\text {V}$ are excluded for the T6ttZG model. For small $m_{\widetilde{\chi }^{0}_{1}}$, gluino masses as large as 1920, 1950, and 1800$\,\text {Ge}\text {V}$ are excluded for the T5qqqqHG, T5bbbbZG, and T5ttttZG models, respectively. Top squark masses as large as 1110$\,\text {Ge}\text {V}$ are excluded for the T6ttZG model. There is close agreement between the observed and expected limits.

## Summary

A search for gluino and top squark pair production is presented, based on a proton–proton collision dataset at a center-of-mass energy of 13$\,\text {Te}\text {V}$ recorded with the CMS detector in 2016. The data correspond to an integrated luminosity of 35.9$\,\text {fb}^{-1}$. Events are required to have at least one isolated photon with transverse momentum ${p_{\mathrm {T}} >100\,\text {Ge}\text {V}}$, two jets with ${p_{\mathrm {T}} >30\,\text {Ge}\text {V}}$ and pseudorapidity ${|\eta |<2.4}$, and missing transverse momentum ${p_{\mathrm {T}} ^\text {miss} >200\,\text {Ge}\text {V}}$.

The data are categorized into 25 exclusive signal regions based on the number of jets, the number of $\mathrm {b}$-tagged jets, and $p_{\mathrm {T}} ^\text {miss}$. Background yields from the standard model processes are predicted using simulation and data control regions. The observed event yields are found to be consistent with expectations from the standard model processes within the uncertainties.

Results are interpreted in the context of simplified models. Four such models are studied, three of which involve gluino pair production and one of which involves top squark pair production. All models assume a gauge-mediated supersymmetry (SUSY) breaking scenario, in which the lightest SUSY particle is a gravitino ($\widetilde{\mathrm {G}}$). We consider scenarios in which the gluino decays to a neutralino $\widetilde{\chi }^{0}_{1}$ and a pair of light-flavor quarks (T5qqqqHG), bottom quarks (T5bbbbZG), or top quarks (T5ttttZG). In the T5qqqqHG model, the $\widetilde{\chi }^{0}_{1}$ decays with equal probability either to a photon and a $\widetilde{\mathrm {G}}$ or to a Higgs boson and a $\widetilde{\mathrm {G}}$. In the T5bbbbZG and T5ttttZG models, the $\widetilde{\chi }^{0}_{1}$ decays with equal probability either to a photon and a $\widetilde{\mathrm {G}}$ or to a $\mathrm {Z}$ boson and a $\widetilde{\mathrm {G}}$. In the top squark pair production model (T6ttZG), top squarks decay to a top quark and $\widetilde{\chi }^{0}_{1}$, and the $\widetilde{\chi }^{0}_{1}$ decays with equal probability either to a photon and a $\widetilde{\mathrm {G}}$ or to a $\mathrm {Z}$ boson and a $\widetilde{\mathrm {G}}$.

Using the cross sections for SUSY pair production calculated at next-to-leading order plus next-to-leading logarithmic accuracy, we place 95% confidence level lower limits on the gluino mass as large as 2120$\,\text {Ge}\text {V}$, depending on the model and the $m_{\widetilde{\chi }^{0}_{1}}$ value, and limits on the top squark mass as large as 1230$\,\text {Ge}\text {V}$, depending on the $m_{\widetilde{\chi }^{0}_{1}}$ value. These results significantly improve upon those from previous searches for SUSY with photons.

## Data Availability

This manuscript has no associated data or the data will not be deposited [Authors’ comment: Release and preservation of data used by the CMS Collaboration as the basis for publications is guided by the CMS policy as written in its document “CMS data preservation, re-use and open access policy” (https://cms-docb.cern.ch/cgi-bin/PublicDocDB/RetrieveFile?docid=6032&filename=CMSDataPolicyV1.2.pdf&version=2 ).]
